# Partnership for Research on Ebola VACcination (PREVAC): protocol of a randomized, double-blind, placebo-controlled phase 2 clinical trial evaluating three vaccine strategies against Ebola in healthy volunteers in four West African countries

**DOI:** 10.1186/s13063-021-05035-9

**Published:** 2021-01-23

**Authors:** Moses Badio, Edouard Lhomme, Mark Kieh, Abdoul Habib Beavogui, Stephen B. Kennedy, Seydou Doumbia, Bailah Leigh, Samba O. Sow, Alpha Diallo, Daniela Fusco, Matthew Kirchoff, Monique Termote, Renaud Vatrinet, Deborah Wentworth, Helène Esperou, H. Clifford Lane, Jerome Pierson, Deborah Watson-Jones, Céline Roy, Eric D’Ortenzio, Brian Greenwood, Genevieve Chêne, Laura Richert, James D. Neaton, Yazdan Yazdanpanah, the PREVAC study team, Coulibaly Abdoulaye, Jamilia Aboulhab, Pauline Akoo, Esther Akpa, Robert Akpata, Sara Albert, Boni Maxime Ale, Benetta C. Andrews, Stephane Anoma, Saw-San Assiandi, Augustin Augier, Ken Awuondo, Moses Badio, Aminata Bagayoko, Nyasha Bakare, Abby Balde, Lamin Molecule Bangura, Kesha Barrington, Eric Barte de Saint Fare, Beth Baseler, Ali Bauder, Claire Bauduin, Luke Bawo, Abdoul Habib Beavogui, Michael Belson, Marion Bererd, Teedoh Beyslow, Blandine Binachon, Julie Blie, Viki Bockstal, Youba Boire, Patricia Boison, Fatorma Bolay, Aliou Boly, Anne Gael Borg, Donna Bowers, Sarah Browne, Barbara Cagniard, Kelly Cahill, Aissata Abdoulaye Camara, Keira Camara, Modet Camara, Cécilia Campion, Jennifer Cash, Siew Pin Chai, Francois Chambelin, Keita Chieck, Geneviève Chêne, Séverine Ciancia, Papa Ndiaga Cisse, Elfrida Clide, Céline Colin, Beth-Ann Coller, Djélikan Siaka Conde, Katherine Cone, Laurie Connor, Nicholas Connor, Joseph Boye Cooper, Sandrine Couffin-Cardiergues, Fatoumata Coulibaly, Mariam Coulibaly, Sandrine Dabakuyo-Yonli, Djeneba Dabitao, Thierry Damerval, Bionca Davis, Gibrilla Fadlu Deen, Eline Dekeyster, Jean-François Delfraissy, Christelle Delmas, Rokia Dembele, Mahamadou Diakite, Alpha Diallo, Mamadou Saliou Diallo, Ayouba Diarra, Oualy Diawara, Bonnie Dighero-kemp, Samba Diop, Waly Diouf, Laurie Doepel, Eric D’Ortenzio, Seydou Doumbia, Moussa Moise Doumbia, Macaya Douoguih, Alain DuChêne, Michael Duvenhage, Risa Eckes Eckes, Avril Egan, Luisa Enria, Hélène Espérou, Cécile Etienne, Allison Eyler, Sylvain Faye, José Fernandez, Suzanne Fleck, Vemy Fofana, Kokulo Franklin, Daniela Fusco, Auguste Gaddah, Marylène Gaignet, Katherine Gallagher, Julia Garcia Gozalbes, Greg Grandits, Maima Gray, Brian Greenwood, Astrid Greijer, Louis Grue, Birgit Grund, Oumar Guindo, Swati Gupta, Fadima Haidara, Benjamin Hamze, Emma Hancox, Gavin Hart, Jean-Christophe Hébert, Esther Heijnen, Patricia Hensley, Lisa Hensley, Elisabeth Higgs, Trudi Hilton, Preston Holley, Marie Hoover, Natasha Howard, Melissa Hughes, Dicko Ilo, Jen Imes, Skip Irvine, David Ishola, Will Jacob, Yvonne Jato, Melvin Johnson, Morrison Jusu, Aboubacar Sidiki Kaba, Myriam Kante, Judith Katoudi, Sakoba Keita, Stephen Kennedy, Babajide Jide Keshinro, Brian Khon, Hassan Kiawu, Mark Kieh, Matt Kirchoff, Mamoudou Kodio, Lamine Koivogui, Tania Kombi, Stacy Kopka, Dickens Kowuors, Christine Lacabaratz, Boris Lacarra, Laurie Lambert, Cliff Lane, Shona Lee, Shelley Lees, Annabelle Lefevre, Bailah Leigh, Frederic Lemarcis, Yves Lévy, Claire Levy-Marchal, Jemilla Lewally, Maarten Leyssen, Edouard Lhomme, Ken Liu, Brett Lowe, Julia Lysander, Claire Madelaine, Ibrah Mahamadou, Daniela Manno, Johnathan Marchand, Siegfried Marynissen, Moses B.F. Massaquoi, Laure Masson, Charly Matard, Onorato Matthew, John McCullough, Noemie Mercier, Pauline Michavila, Tracey Miller, Alejandra Miranda, Soumaya Mohamed, Tom Mooney, Hans Morsch, Dally Muamba, Rita Lukoo Ndamenyaa, James Neaton, Désiré Neboua, Micki Nelson, Kevin Newell, Vinh-kim Nguyen, Leslie Nielsen, Millimouno Niouma, Kim Offergeld, Matthew Onorato, Uma Onwuchekwa, Susan Orsega, Inmaculada Ortega-Perez, Cynthia Osborne, Tuda Otieno, Sushma Patel, Nathan Peiffer-Smadj, Robert Phillips, Jerome Pierson, Peter Piot, Micheal Piziali, Stephany Pong, Calvin Proffitt, Alexandre Quach, Corina Ramers-verhoeven, Nadeeka Randunu, Laura Richert, Priscille Rivière, Cynthia Robinson, Griet Van Roey, Céline Roy, Amy Falk Russell, Mohamed Samai, Sibiry Samake, Ballan Sangare, Ibrahim Sanogo, Yeya Sadio Sarro, Sadio Sarro, Mélanie Saville, Serge Sawadogo, Maxime Schvartz, Christine Schwimmer, Fatou Secka, Jacques Seraphin, Denise Shelley, Sophia Siddiqui, Jakub Simon, Shelly Simpson, Billy Muyisa Sivahera, Irvine Skip, Karen Slater, Mary Smolskis, Elizabeth Smout, Emily Snowden, Anne-Aygline Soutthiphong, Samba Sow, Ydrissa Sow, Daniel Splinter, Simone Spreng, Helen Stapleton, Jeroen Stoop, Mary Sweeney, Sienneh Tamba, Mili Tapia, Jemee Tegli, Monique Termote, Rodolphe Thiebaut, Greg Thompson, John Tierney, Abdoulaye Touré, Stacey Traina, Awa Traore, Moussa Traore, Tijili Tyee, David Vallée, Katrien V Van Der Donck, Renaud Vatrinet, Nadia Verbruggen, Corine Vincent, Susan Vogel, Cedrick Wallet, Deborah Watson-Jones, Deborah Wentworth, Cecelia Wesseh, Jimmy Whitworth, Aurelie Wiedemann, Wouter Willems, Julian Williams, Barthalomew Wilson, Njoh Wissedi, Jayanthi Wolf, Ian Woods, Alie Wurie, Delphine Yamadjako, Marcel Yaradouno, Yazdan Yazdanpanah, Zara Zeggani

**Affiliations:** 1 Partnership for Research on Ebola Virus in Liberia (PREVAIL), Monrovia, Liberia; 2 Univ. Bordeaux, Inserm, Bordeaux Population Health Research Center, UMR 1219, CHU Bordeaux, CIC 1401, EUCLID/F-CRIN Clinical Trials Platform, F-33000 Bordeaux, France; 3 Centre National de Formation et de Recherche en Santé Rurale de Mafèrinyah, Mafèrinyah, Guinea; 4 University of Sciences, Technique and Technology of Bamako, Bamako, Mali; 5 College of Medicine and Allied Health Sciences (COMAHS), University of Sierra Leone, Freetown, Sierra Leone; 6 Centre pour le Développement des Vaccins, Bamako, Mali; 7 INSERM, Pôle de Recherche Clinique, 75013 Paris, France; 8 National Institute of Allergy and Infectious Diseases, Bethesda, MD USA; 9 REACTing, Institut Thématique Immunologie, Inflammation, Infectiologie et Microbiologie, Inserm, Paris, France; 10 Division of Biostatistics, School of Public Health, University of Minnesota, Minneapolis, MN USA; 11 London School of Hygiene & Tropical Medicine, London, UK; 12 AP-HP, Hôpital Bichat-Claude Bernard, Service de Maladies Infectieuses et Tropicales, F-75018 Paris, France

**Keywords:** Ebola, Vaccine, Clinical trials, Protocol, Randomized controlled trials

## Abstract

**Introduction:**

The Ebola virus disease (EVD) outbreak in 2014–2016 in West Africa was the largest on record and provided an opportunity for large clinical trials and accelerated efforts to develop an effective and safe preventative vaccine. Multiple questions regarding the safety, immunogenicity, and efficacy of EVD vaccines remain unanswered. To address these gaps in the evidence base, the Partnership for Research on Ebola Vaccines (PREVAC) trial was designed. This paper describes the design, methods, and baseline results of the PREVAC trial and discusses challenges that led to different protocol amendments.

**Methods:**

This is a randomized, double-blind, placebo-controlled phase 2 clinical trial of three vaccine strategies against the Ebola virus in healthy volunteers 1 year of age and above. The three vaccine strategies being studied are the rVSVΔG-ZEBOV-GP vaccine, with and without a booster dose at 56 days, and the Ad26.ZEBOV,MVA-FN-Filo vaccine regimen with Ad26.ZEBOV given as the first dose and the MVA-FN-Filo vaccination given 56 days later. There have been 4 versions of the protocol with those enrolled in Version 4.0 comprising the primary analysis cohort. The primary endpoint is based on the antibody titer against the Ebola virus surface glycoprotein measured 12 months following the final injection.

**Results:**

From April 2017 to December 2018, a total of 5002 volunteers were screened and 4789 enrolled. Participants were enrolled at 6 sites in four countries (Guinea, Liberia, Sierra Leone, and Mali). Of the 4789 participants, 2560 (53%) were adults and 2229 (47%) were children. Those < 18 years of age included 549 (12%) aged 1 to 4 years, 750 (16%) 5 to 11 years, and 930 (19%) aged 12–17 years. At baseline, the median (25th, 75th percentile) antibody titer to Ebola virus glycoprotein for 1090 participants was 72 (50, 116) EU/mL.

**Discussion:**

The PREVAC trial is evaluating—placebo-controlled—two promising Ebola candidate vaccines in advanced stages of development. The results will address unanswered questions related to short- and long-term safety and immunogenicity for three vaccine strategies in adults and children.

**Trial registration:**

ClinicalTrials.gov NCT02876328. Registered on 23 August 2016.

**Supplementary Information:**

The online version contains supplementary material available at 10.1186/s13063-021-05035-9.

## Introduction

The Ebola virus disease (EVD) outbreak in 2014–2016 in West Africa was the largest since the discovery of the virus in 1976 with more than 28,000 confirmed cases of EVD and 11,000 deaths in Guinea, Liberia, and Sierra Leone (ref: WHO Ebola situation report 2016). The West-African outbreak prompted the rapid clinical evaluation of vaccine and therapeutic candidates that were in early development. As evidenced by the ongoing Ebola outbreak in the Democratic Republic of Congo (DRC) ongoing since August 2018, along with other public health measures, efforts to develop an effective and safe vaccine against Ebola virus disease must continue [1].

By the end of 2015, new cases of EVD in West Africa had dramatically decreased. An open-label, cluster-randomized, ring vaccination trial conducted in Guinea, the “Ebola ca suffit” trial, randomized contacts and contacts of contacts to receive the Merck/New Link rVSV∆G-ZEBOV-GP vaccine immediately after randomization or 21 days later. There were no cases of Ebola virus disease 10 days or more following vaccination among 2108 contacts and contacts of contacts who were vaccinated immediately compared to 10 such cases in 1429 contacts and contacts of contacts who initially consented to receive vaccination 21 days after randomization [2]. In that trial, which was conducted mainly in adults, no EVD events occurred more than 10 days after vaccination. Based upon these and other data this vaccine has been approved by the European Medicines Agency (EMA) and the U.S. Food and Drug Administration (FDA) for the prevention of Ebola virus disease in individuals 18 years of age and older. No immunogenicity and limited safety data were collected in the “Ebola ca suffit” trial, especially in children. Two other vaccine strategies, the single-dose GlaxoSmithKline (GSK) ChAd3-EBO Z (replication-deficient Chimpanzee adenovirus type 3-derived vector encoding the Ebola virus Zaire [EBO Z] GP) vaccine and the Johnson & Johnson (J&J) 2-dose heterologous vaccination regimen, Ad26.ZEBOV,MVA-FN-Filo, have completed phase 2 testing. The heterologous two-dose Ad.26.ZEBOV,MVA-BN-Filo vaccine regimen has shown an acceptable safety profile and was well-tolerated and immunogenic in healthy 18–50 year-old adults volunteers from France and England [3, 4] and Africa countries [5, 6]. Data on safety, tolerability, and immunogenicity from multiple studies supported the European marketing authorization for this Ebola two-dose heterologous vaccine regimen granted by the European Commission after assessment by European Medical Agency in July 2020 for the prevention of Ebola virus disease caused by the Zaire ebolavirus species in individuals aged one year and above. In addition, safety and immunogenicity up to 12 months after vaccination of the rVSV∆G-ZEBOV-GP and ChAd3-EBO Z vaccine have been evaluated in a phase 2, placebo-controlled trial of adults in Liberia (Partnership for Research on Ebola Virus in Liberia I [PREVAIL I]) [7]. The planned phase 3 component of PREVAIL I could not be completed because of the decline in cases of EVD resulting from public health efforts.

While there was substantial progress in the development of Ebola vaccines during the 2014–2016 West African epidemic, multiple questions regarding the safety and efficacy of EVD vaccines remain unanswered, including the durability and the immediacy of immune responses generated by different vaccine strategies with and without a booster, and the safety of these Ebola vaccines in special populations, particularly children.

To address these gaps, a phase 2 trial, the Partnership for Research on Ebola Vaccines (PREVAC) trial, was designed and initiated in 2017 to compare three vaccine strategies with placebo in adults and children in Guinea, Liberia, Mali, and Sierra Leone.

## Methods

### Trial design

The PREVAC trial is a randomized, double-blind, superiority, placebo-controlled phase II clinical trial evaluating three vaccine strategies against the Ebola virus.

We have conducted this trial as per the recommendations for interventional trials (SPIRIT). The final reporting of this trial will be in accordance with the Consolidated Standards of Reporting Trials (CONSORT) statement.

### Trial location

Healthy volunteers were enrolled at 6 sites in four countries: Guinea at two sites (Landreah located in an urban area in Conakry and Maferinyah, a rural area in the Forecariah region), Liberia (Redemption Hospital in Monrovia), Mali at two sites (Center for Vaccine Development (CVD) and the University Clinical Research Center (UCRC), both in the capital Bamako), and Sierra Leone (Mambolo, a rural community in Kambia District, northern Sierra Leone).

### Eligibility criteria

Inclusion into the study was based on the following criteria: (1) willingness to participate and sign informed consent/assent, (2) age ≥ 1 year, (3) planned residency in the area of the study site for the next 12 months, and (4) willingness to comply with the protocol requirements.

Participants were excluded from enrolment based on the following: (1) fever > 38 °C, (2) history of EVD (self-report), (3) pregnancy (a negative urine pregnancy test was required for females of child-bearing potential), (4) positive HIV test for participants < 18 years of age, (5) reported current breast-feeding, (6) prior vaccination against Ebola, (7) any vaccination in the past 28 days or planned within the 28 days after randomization, and (8) in the judgment of the clinician, any clinically significant acute/chronic condition that would limit the ability of the participant to meet the requirements of the study protocol.

### Objectives

The primary aim of the study is to evaluate the durability and immediacy of the antibody response to vaccination. The primary objective of the trial is to compare each of the three vaccine strategies with the pooled placebo group (3 pair-wise comparisons) for antibody responses 12 months after randomization (durability of response). This will be addressed separately for adults and children. Other objectives stated in the protocol are included in Additional file 1: Appendix 1.

For the pharmaceutical companies providing the vaccines, information is also being collected to support regulatory filings.

### Interventions

The three vaccine strategies being studied are the rVSVΔG-ZEBOV-GP vaccine, with and without a booster dose at 56 days, and the 2-dose heterologous vaccination regimen Ad26/MVA. One (1) mL from a 3 mL syringe of the rVSVΔG-ZEBOV-GP vaccine was administered for the prime and booster vaccination. The Ad26/MVA requires a 0.5-mL administration of Ad26.ZEBOV from a 3-mL syringe for the first vaccination dose and second dose vaccination of MVA-BN-Filo (0.5 mL from a 3-mL syringe) at 56 days.

The 2-dose heterologous vaccination regimen Ad26.ZEBOV,MVA-BN-Filo (0.5 mL from a 3 mL syringe for both doses) is comprised of an Ad26.ZEBOV vaccine which consists of a single recombinant, replication-incompetent human Ad26 vector, constructed to express the Ebola virus Mayinga GP. The second dose with MVA-BN-Filo at 56 days encodes the GP of Sudan virus (SUDV; formerly known as Ebola Virus Sudan), EBOV (formerly known as Ebola Virus Zaire), and Marburg Virus (MARV) Musoke and the nucleoprotein of Tai Forest virus (TAFV; formerly known as Côte d’Ivoire ebolavirus). The Ad26.ZEBOV,MVA-BN-Filo vaccination regimen was given at the same dose in versions 2.0, 3.0, and 4.0 of the PREVAC protocol. Version 1.0 was never implemented.

The rVSVΔG-ZEBOV-GP vaccine is comprised of a single rVSV isolate (11,481 nt) modified to replace the gene encoding the VSV G envelope GP with the gene encoding the envelope GP from ZEBOV (Kikwit, 1995 strain) (1 mL IM administration). The rVSVΔG-ZEBOV-GP vaccine was not used in Version 2.0. The rVSVΔG-ZEBOV-GP dose was given as a 2-fold diluted dose (approximately 5 × 10^7^ plaque-forming units [pfu]/mL) in Version 3.0 and was given as an undiluted dose (geometric mean of available assays 9.4 × 10^7^ pfu/mL) in Version 4.0. The doses of rVSVΔG-ZEBOV-GP used in versions 3.0 and 4.0 are referred to as the diluted and undiluted doses, respectively.

The placebo is sterile normal saline (sodium chloride 0.9% for injection, United States Pharmacopeia, preservative-free).

### Study setting

**Fig. 1:**
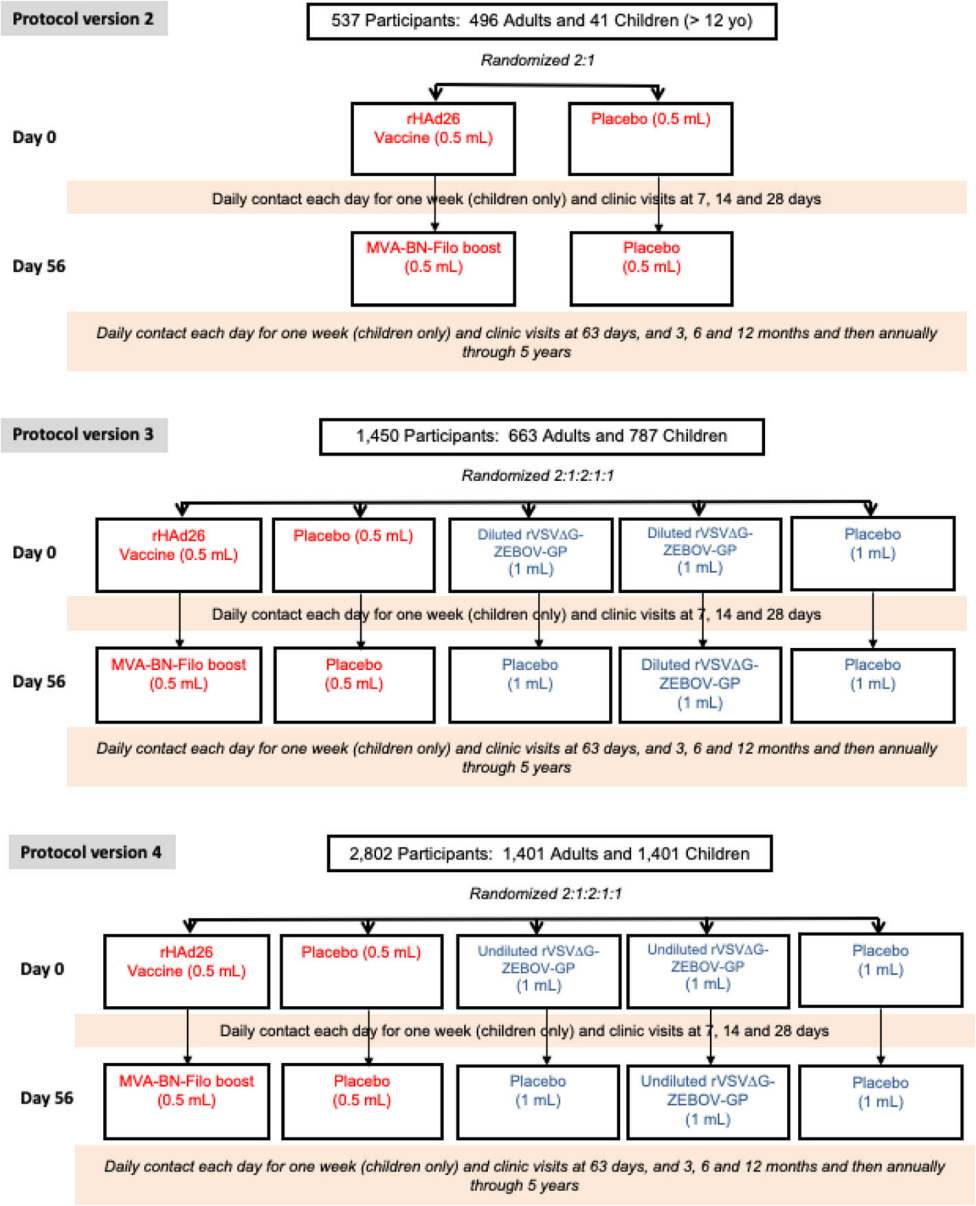
PREVAC study design for each version of the protocol

Initially, it was envisaged that eligible participants would be randomized to one of the following five groups in a 2:1:2:1:1 allocation: (1) Ad26.ZEBOV (prime vaccination at day 0) (0.5 mL) followed by a second dose with MVA-BN-Filo (0.5 mL) at 56 days, (2) placebo (at randomization and at 56 days) (0.5 mL), (3) rVSV∆G-ZEBOV-GP (prime at day 0) (1 mL) followed by placebo boost (1 mL) at 56 days, (4) rVSV∆G-ZEBOV-GP (prime at day 0) (1 mL) followed by rVSV∆GZEBOV-GP booster dose (1 mL) at 56 days, and (5) normal saline placebo (prime at day 0 and boost at 56 days) (1 mL) (Fig. 1). The study design included two placebo groups because the Ad26.ZEBOV and rVSV∆G-ZEBOV-GP vaccines were administered at different volumes and therefore each required a different placebo to match their specific volume of injection. For the primary analyses, the two placebo groups will be pooled.

A week prior to the commencement of enrollment for version 1.0 of the PREVAC trial protocol, an ongoing open-label study (the PREPARE study; ClinicalTrials.gov Identifier: NCT02788227) evaluating the safety and immunogenicity of the rVSV∆G-ZEBOV-GP vaccine among healthcare workers in the USA and Canada was temporary paused because 3 out of the 9 initial participants who had received the rVSV∆G-ZEBOV-GP vaccine reported arthritis. A vaccine lot investigation was undertaken which resulted in no quality findings. Due to site readiness in both Guinea and Liberia, while the lot investigation was ongoing, the protocol was revised to version 2.0 to exclude the rVSV∆G-ZEBOV-GP and matching placebo arm. Thus, the PREVAC trial commenced with a two-arm strategy and randomized participants ≥ 12 years of age to the Ad26.ZEBOV first dose (0.5 mL) followed by an MVA-BN-Filo second dose (0.5 mL) at 56 days or to matching placebo in a 2:1 randomization ratio (Fig. 1—top panel). Version 2.0 aimed to enroll up to 600 participants to allow migration quickly to the original five arm randomization.

Even though no quality finding was found in the initial lot of rVSV∆G-ZEBOV-GP vaccine, a new lot was prepared. Upon review of the certificate of analysis of this new lot (release potency of 9.4 × 10^7^ pfu/mL), it was decided to initially give the rVSV∆G-ZEBOV-GP vaccine at a 2-fold dilution (approximately 5 × 10^7^ pfu/mL) which the study team referred to as the diluted dose. In June 2017, the protocol was amended and version 3.0 included randomization to 5 groups with the rVSV∆G-ZEBOV-GP vaccine groups being given the diluted dose (Fig. 1—middle panel). As a safety precaution, enrollment was staggered by age group, starting with children aged 12–17 and adults. After 70 children aged 12–17 had been enrolled, safety data until day 28 were reviewed by an independent Data and Safety Monitoring Board (DSMB), which identified no safety concerns and recommended opening enrollment to children aged 5–11 years. The procedure was repeated for the 5–11 and 1–4 age groups. In each age group, a DSMB review was conducted after 70 children were enrolled and followed for 28 days, before enrollment was opened to the next younger age group. It was decided that once the safety of the diluted dose of rVSV∆G-ZEBOV-GP had been established in children, the trial protocol would be amended to use the undiluted rVSV∆G-ZEBOV-GP vaccine.

In April 2018, after the safety of all three vaccine strategies, including the diluted dose of rVSV∆G-ZEBOV-GP, had been determined in each of three age groups of children (1–4, 5–11, and 12–17 years) by the independent DSMB, the PREVAC protocol was again amended. This version of PREVAC (version 4.0) follows the design originally planned (version 1.0) and that of version 3.0 except that the undiluted dose of the rVSV∆G-ZEBOV-GP vaccine (9.4 × 10^7^ pfu/mL) was to be used (Fig. 1—bottom panel). The enrollment targets in version 4.0 were 1400 children aged 1–17 years and 1400 adults. The investigators of PREVAC agreed that the primary results would be based on data from version 4.0.

Both versions 2.0 and 3.0 of the study protocols were implemented in Guinea at Landreah and Maferinyah sites and at Redemption Hospital in Liberia. Protocol version 4.0 was implemented in these three sites and also in the CVC and UCRC sites in Mali and at the Mambolo site in Sierra Leone.

### Randomization and blinding

The randomization groups and allocation ratio for each version of the protocol are presented in Fig. 1. For each vaccination center, the randomization schedule was prepared centrally, using block randomization to ensure the desired allocation ratio for each vaccination center.

Syringes were prepared one by one (whenever a participant was ready to be randomized) at the local study pharmacies by unblinded staff not involved in any other trial procedure. The syringe content (type of vaccine or placebo) was defined according to a central list that had been prepared by the University of Minnesota prior to the start of the trial, reflecting the above mentioned specifications for the randomization schedule. Allocation concealment was achieved by the use of a computer application that only revealed the next entry on the list one by one to the on-site pharmacy staff. The prepared syringe was labeled with a unique Syringe IDentifier (SID) and a bar code identifier tear-off label. To ensure blinding of the remaining site staff and the trial participants, syringe content was masked by a label covering the whole syringe. Although it cannot be excluded that the staff administering the vaccine could distinguish between the syringes containing 0.5 mL or 1 mL, they did not know whether the syringe contained active vaccine or placebo. At the time of vaccination, the tear-off label on the syringe with the SID was attached to the baseline case report form (CRF) creating the primary link between the vaccine administered and the participant identification (PID). This event is considered the point of randomization and ensures that all randomized participants receive an injection, active or placebo. The syringes for the booster vaccination at day 56 were prepared at the local pharmacies, filled with an active vaccine or placebo depending on the participant’s random assignment, and labeled with the PID. The laboratories carrying out the safety and immunogenicity analyses were blinded to the random assignment. Study participants and clinical staff assessing the study participants for safety and laboratory outcomes will remain fully blinded until all participants from the three protocol versions have completed 12 months of follow-up and results become available. Procedures were put into place to break the blind if necessitated by a medical emergency. If unblinding of a participant for safety reasons was required, it was documented.

### Study endpoints

The primary endpoint is the GP-EBOV antibody response 12 months after randomization. This is the primary endpoint that will be used to compare the immunogenicity of the three vaccine strategies with placebo. Other endpoints are as follows;

- For rVSV∆GZEBOV-GP arms only, GP-EBOV antibody response at day 28 after vaccination will be used for regulatory purposes for comparison to other studies and for bridging children to adults.
- For the Ad26.ZEBOV,MVA-BN-Filo vaccine arm only, GP-EBOV antibody response at month 3 after randomization (approximately 28 days after the second dose of vaccine) will be used for regulatory purposes.

The primary analysis will be performed separately, for adults and children, and will exclude participants with elevated antibody titers at baseline.

Antibodies to the Ebola virus GP will be measured with the Filovirus Animal Nonclinical Group (FANG) ELISA assay [8]. The precise definition of antibody responders will be defined prior to unblinding of the study results. Other assays may also be used. If a correlate of protection is identified, stored sera will be used to measure the correlate and carry out comparisons of the three vaccine strategies with placebo and with one another.

### Data collection plan

Time and events schedule for baseline and regularly scheduled follow-up visits in the PREVAC study is described in Table 1. After it had been established that volunteers met the eligibility criteria and informed consent had been obtained from them or, in the case of children, a parent or guardian background data were collected. Demographics and a short medical history were obtained, blood was drawn as specified by the protocol, and then participants then received their first dose of the vaccine (“prime vaccination”). Randomization occurred at the point of vaccination as described above. For 30 min after the vaccination, participants were watched closely, injection site reactions and targeted symptoms were assessed, and possible grade 3 or 4 adverse events were recorded.

**Table 1:** Time and event schedule for baseline and regularly scheduled follow-up visits in the PREVAC study

Events	Adults	Children
**Screening** (children must have a documented negative HIV test prior to randomization)		X
**Baseline**		
Informed consent/assent	X	X
Initial vaccination	X	X
Demographics ^a)^	X	X
Contact information ^b)^	X	X
Indicators of increased risk ^c)^	X	X
Height, weight and temperature	X	X
Mid-upper arm circumference for children 1–5 years		X
Pregnancy test for females of child-bearing potential, i.e., females who have experienced menarche or who are aged 14 years and older	X	X
Blood sample for immunogenicity testing and future research	X	X
Blood sample for chemistries and CBC	X	X
Blood sample for HIV/syphilis testing	X	X
Saliva sample for assessing viral shedding (subsample)		X
Stored blood for assessing T cell and memory B cell function in a subset of participants in Guinea	X	
Injection site reactions, targeted symptoms of any grade severity, and grade 3 or 4 AEs following prime vaccination	X	X
HIV pre-counseling	X	X
**Days 1 to 6 following prime and booster vaccination**		
Contact for injection site reactions, targeted symptoms of any grade severity, grade 3 or 4 AEs, measurement of temperature, and possible SAEs in children		X
**Day 7 and day 63**		
Injection site reactions, targeted symptoms of any grade severity, and grade 3 or 4 AEs	X	X
Blood sample for immunogenicity testing and future research	X	X
Saliva sample for assessing viral shedding (subsample)		X
Blood sample for assessing T cell and memory B cell function in a subset of adults in Guinea	X	
HIV and syphilis post-counseling referral	X	X
Blood sample for chemistries and CBC		X
Temperature	*X*	X
**Day 14**		
Injection site reactions, targeted symptoms of any grade severity, and grade 3 or 4 AEs	X	X
Temperature	X	X
Blood sample for immunogenicity testing and future research	X	X
Saliva sample for assessing viral shedding (subsample)		X
Blood sample for assessing T cell and memory B Cell function in a subset of adults in Guinea	X	
**Day 28**		
Injection site reactions, targeted symptoms of any grade severity, and grade 3 or 4 AEs	X	X
Temperature	X	X
Weight		X
Blood sample for immunogenicity testing and future research	X	X
Saliva sample for assessing viral shedding (subsample)		X
**Day 56**		
Booster vaccination	X	X
Blood sample for immunogenicity testing and future research	X	X
Pregnancy test for females of childbearing potential, i.e., females who have experienced menarche or who are aged 14 years and older	X	X
Temperature	X	X
Injection site reactions, targeted symptoms of any grade severity, and grade 3 or 4 AEs following booster vaccination	X	X
Saliva sample for assessing viral shedding (subsample)		X
Blood sample for assessing T cell and memory B cell function in a subset of adults in Guinea	X	
**Day 70**		
Blood sample for assessing T cell and memory B cell function in a subset of adults in Guinea	X	
**Months 3 and 6**		
Injection site reactions, targeted symptoms of any grade severity, and grade 3 or 4 AEs (Month 3 only)	X	X
Temperature	X	X
Weight		X
Mid-upper arm circumference for children 1–5 years		X
Blood sample for immunogenicity testing and future research	X	X
Saliva sample for assessing viral shedding (subsample at month 3 only)		X
**Months 12, 24, 36, 48, and 60**		
Temperature	X	X
Weight		X
Height		X
Mid-upper arm circumference for children 1–5 years		X
Blood sample for immunogenicity testing and future research	X	X
Survey data for the study on barriers and facilitators to PREVAC retention	X	X
Blood sample for the parasitology assays for the malaria substudy in Sierra Leone and in Guinea (Landreah site)	X	X
Blood sample for assessing T cell and memory B cell function in a subset of adults in Guinea	X	
**SAEs throughout follow-up**	X	X
**EVD events (reported as soon as aware) throughout follow-up and where possible a stored blood sample for future research**	X	X
**Death (reported as soon as aware) throughout follow-up**	X	X
**Pregnancy outcome for women who become pregnant in the first 3 months of follow-up after prime vaccination**	X	X

^a)^Birth month and year, gender

^b)^Contact information for self and 2 contacts who will know how to locate the volunteer

^c)^Role as health care worker, contact with persons known to have EVD

After the prime vaccination at study entry, initial follow-up visits occurred at: 7 (± 3 days), 14 (± 3 days), and 28 (± 7 days) days. The booster dose of vaccine was administered on day 56 (53 to 66 days) with further follow-up visits at 63 days (7 ± 3 days after the booster vaccination), at 3 months (± 14 days), 6 months (± 1 month), and 12 months (± 1 month). Visits will continue at 24 (± 6 month), 36 (± 6 month), 48 (± 6 month), and 60 (− 6 month; + 1 month) months as part of the PREVAC-UP study. At each follow-up visit up to month 3, injection site reactions, targeted symptoms, and grade 3 or 4 adverse events that occurred since the previous visit are reported. At all follow-up visits, malaria and serious adverse events (SAEs) are reported, the temperature is recorded, and blood is drawn and stored for future immunogenicity assessments and other research.

For children, additional data were collected. Blood chemistries were assessed prior to the prime and booster vaccination at baseline and day 56 respectively and 7 days after each vaccination. Also, during the first week following the prime and booster vaccination, daily contacts were made with children to assess injection site reactions, targeted symptoms and serious adverse events (SAEs), and body temperature.

Blood samples are collected at each site and processed according to their final use. A local laboratory analyzed blood samples at day 0 for adults and at days 0, 7, and 63 for children and adolescents for blood chemistry and hematology to assess potential vaccine toxicities. Laboratory values are graded for severity according to the Division of AIDS (DAIDS) Adverse Event Grading tables. Serology for HIV and syphilis was performed prior to randomization using rapid tests. If the participant had a positive HIV or syphilis test, he/she was offered post-counseling and was referred to a pre-identified health structure for treatment. All countries use their own normal ranges as a reference. For Guinea, the Ghana values from Dooso et al. [9, 10] were used; for Liberia, those used in PREVAIL 1 [7] were adopted; for Sierra Leone, they were those used for the EBL3001 trial (NCT02509494); and for Mali, the normal values used were those from Khone et al. [11].

The FANG assay to measure the antibody response to Ebola glycoprotein is performed at the Liberian Institute for Biomedical Research (LIBR) laboratory in Monrovia for participants from Guinea and Sierra Leone, at the UCRC for participants from Mali, and at the NIAID Integrated Research Facility (IRF) (Maryland, USA) for participants from Liberia. Aliquots are shipped from sites to the relevant laboratory at regular intervals. Quality controls for intra- and inter-laboratory reproducibility are performed on a regular basis. Additional antibody response testing using a validated FANG assay will be performed for specific time-points (day 0, day 28, month 3, and month 12) at Quest diagnostics Clinical Laboratories Inc., San Juan Capistrano, USA (FOCUS), as part of the regulatory activities of Merck and JnJ.

A CRF with a unique PID is completed for each participant consented and screened. The CRF is an eCRF developed with Ennov Clinical in English and French. Due to logistical hurdles, direct electronic data collection was not an option. Therefore, the clinical data are collected on paper CRF Forms and then entered in the eCRF the same day. Laboratory results conducted by the study team are directly extracted for the local Laboratory information management system and transferred on a daily basis to the centralized data center (EUCLID/FCRIN Clinical Trials Platform, Inserm/Univ Bordeaux, France).

Source data verification was done by regular on-site monitoring under the responsibility of the sponsors on site to ensure data integrity, and moreover, remote monitoring was done in almost real time to identify trends and potential problems that go beyond just transcription problems from source documents.

### Sample size and statistical analysis

The sample size for version 4.0 of the protocol was calculated to provide power to compare safety and immunogenicity separately for adults (*N* = 1400) and children (*N* = 1400). The sample size is greater than that which is required to address the primary objectives because, if a correlate of protection is identified, the vaccine strategies will be compared with one another for that correlate using an intention to treat analysis. Expected differences between vaccine groups may be smaller than comparisons with placebo and the correlate may have greater variability than the assay which will be used to measure antibody titers to address the primary, secondary, and exploratory objectives. The larger sample size will also permit the exploration of subgroups and preserve power in the event there are more participants with elevated antibodies at baseline than anticipated.

With a type 1 error = 0.0167 (2-sided) to adjust for the three comparisons, separately for adults and for children in all age groups combined, and power = 0.90, even if the percent with a positive antibody response at 12 months is 50% in a vaccine group, with equal allocation, approximately 30 participants per group (60 participants in total) are needed assuming the percent in the placebo group with a positive antibody response is approximately 7%. With unequal allocation as for the rVSVΔG-ZEBOV-GP with the booster versus placebo comparison, a total of 63 participants (21 vaccinated with rVSVΔG-ZEBOV-GP with a boost and 42 vaccinated with placebo), a difference of 50% versus 7% can also be detected at 12 months with 87% power. These sample size estimates indicate that power for the planned subgroup analysis by age is also appropriate.

The planned sample size is also adequate for the comparisons with placebo if more than 4% of participants are antibody positive at baseline and excluded from the primary analysis and if there are some missing data at 12 months of follow-up.

More information about the statistical analysis of the PREVAC trial is described in Additional file 1: Appendix 2.

### Substudies

There are currently two substudies ongoing and two planned in PREVAC. The immunological substudy is being conducted in Guinea with the primary objective of evaluating the T cell responses induced by the two vaccines candidates (rVSV∆G-ZEBOV-GP and Ad26.ZEBOV/MVA-BN-Filo) in adults and their persistence until 1 year after vaccination and longer (middle/long term). The second substudy, being conducted in Liberia, is investigating viral shedding of the rVSV∆G-ZEBOV-GP vaccine. The objective of this sub-study is to estimate the proportion of children who shed vaccine virus and to quantify the rVSV∆G-ZEBOV-GP vaccine shedding in children (participants aged < 18 years) after the prime and boost vaccinations. A third sub-study, assessing the impact of malaria infections on immune responses to the vaccines, is planned to take place in Sierra Leone and Guinea. A fourth sub-study, aiming to identify multi-level determinants of participant retention in the PREVAC trial to develop recommendations that will benefit future clinical research in resource limited settings affected by Ebola and other infectious diseases, is planned to take place in the four countries.

### Trial governance

The Partnership for Research on Ebola Vaccinations (PREVAC) was established as an international consortium at the end of the West-African outbreak in 2015 to focus on Ebola research activities and to prevent or respond effectively to the next potential Ebola outbreak. The consortium includes research and academic institutions (the French Institute for Health and Medical Research [Inserm], the London School of Hygiene & Tropical Medicine [LSHTM], the US National Institutes of Health [NIH], the Universities of Bordeaux and Minnesota), health authorities and scientists from four Ebola-affected countries (Guinea, Liberia, Sierra Leone, and Mali), non-governmental organizations (the Alliance for International Medical Action and Leidos Biomedical Research, Inc.), and pharmaceutical companies (MSD, Janssen Vaccines & Prevention B.V., and Bavarian Nordic). Members of the PREVAC study group conducting the vaccine trial are listed in Additional file 1: Appendix 3.

Trial governance is detailed in Additional file 1: Appendix 4 - Figure 1. The trial is being conducted under the direction of a Trial Steering Committee (TSC), which provides overall supervision for the trial on behalf of the three sponsors (Inserm, LSHTM, NIH) and the host countries. Members of the TSC (Additional file 1: Appendix 4) are blinded to interim safety and immunogenicity results.

An Executive Committee is composed of representatives of the sponsors and the coordinating investigator. Ad hoc meetings of the executive committee are organized when a sponsor-level discussion is needed. The executive committee also has the final say in the event consensus is not reached between TSC members.

A DSMB provides independent, expert oversight for the trial and monitors accumulating safety data for adults and children in each age group (1–4, 5–11, and 12–17 years) using reports provided by unblinded statisticians from the University of Minnesota (Division of Biostatistics, Minneapolis, USA).

The day-to-day operations and management of the trial are coordinated by the Trial Management Team (TMT). Daily monitoring of inclusions and retention rates are carried out in the central database, and blind reports are made available to the TMT via a secure website by a blinded statistician from EUCLID/FCRIN Clinical Trials Platform (Inserm/Univ Bordeaux, France). In coordination with the sites, this close monitoring has helped to achieve approximately the same number of children in each age group and a gender balance in both children and adults.

A centralized pharmacovigilance service has been implemented for trial safety management. Adverse events (AEs) occurring in enrolled participants are reported by investigators as soon as they become aware of them. All serious adverse events (SAEs) are reported to the sponsors immediately and no later than 24 h after the investigator becomes aware of them. The medical officer (MO) responsible for pharmacovigilance firsts performs a partial unblinding of a potential suspected unexpected serious adverse reaction (SUSAR) to assess whether the SAR is unexpected for either product. If the event is unexpected for the product arm assigned (vaccine or matched placebo), the MO asks to be fully unblinded to determine whether the participant received active vaccine or placebo. This full unblinding is also performed via the web-based unblinding application monitored by the University of Minnesota. All SUSARs or other new safety data that would constitute new information for competent authorities are reported to the DSMB. The DSMB may pause enrolment in the event of vaccine-related deaths or SAEs that are considered vaccine-related. The DSMB may also pause enrolment or request that participants be notified if there is an increased frequency of unanticipated adverse effects.

### Ethical and regulatory aspects

During the enrollment process, there was widespread communication with local communities about the trial. Potential participants joined a detailed group information session prior to an individual session that included the signing of an informed consent form if they agreed to enroll. A flip chart describing the study was used to ensure that illiterate volunteers and minors understood the study requirements and risks and benefits in the presence of an impartial witness. In Liberia and Sierra Leone, minors aged 7–17 years signed an informed assent form after their parents/guardian provided consent for their participation in the study. Assent was not required in either Guinea or Mali. Minors who declined participation in the study after reviewing the assent materials were not enrolled even if their parent(s) or legal guardian consented to their participation.

The study protocol, the informed consent and assent forms (Additional file 1: Appendix 5), including participants’ information materials, were approved by ethics committees of the sponsors (INSERM IRB 00003888, LSHTM) and the implementing countries (Guinea, Liberia, Mali, and Sierra Leone) before each version of the protocol was implemented. NIH established an institutional reliance agreement with INSERM to rely upon the INSERM ethics committee. The study is registered at www.ClinicalTrials.gov (NCT02876328) and in the Pan African Clinical Trials Registry (PACTR201712002760250).

The data of this trial will be disseminated through national and international conferences and peer-reviewed publications.

### Social mobilization

Community-level social mobilization activities were developed in each site to engage local populations in interventional research and thus facilitate study enrollment. Site-level procedures were also developed to support participant retention, including visit incentives, participant tracking, digital and community-based outreach, and use of community champions and community mobilization techniques.

## Results

### Enrollment rate and retention

From April 2017 to December 2018, six sites in the four collaborating countries (Guinea, Liberia, Mali, and Sierra Leone) screened 5002 subjects and enrolled 4789 participants in one or other version of the study protocol (Additional file 1: Appendix 6 - Figure 1-3, Appendix 7). A total of 537 participants were enrolled in version 2.0 of the protocol from April to July 2017 in Guinea and Liberia. A total of 1450 participants were enrolled in version 3.0 of the protocol from July 2017 to March 2018 in Guinea and Liberia. Enrollment in version 4.0 occurred over a period of eight months (April to December 2018) with a total of 2802 participants enrolling in the six sites in the four countries, including 1401 children with an equal distribution between the three age groups. An average of 35.8, 41.4, and 79.2 participants per week were enrolled in version 2.0, 3.0, and 4.0 respectively.

For protocol version 4.0, the proportion of visits attended at M12 was 95.8% whilst for protocol versions 2.0 and 3.0, it was 96.1% and 97.1% respectively.

### Baseline characteristics

The baseline characteristics of the participants included in all versions of the PREVAC trial are described in Table 2. Overall, the median age was 18 years [IQR 11–28] and females constituted 45.5% of the study population. Of the 4789 participants, 2560 (53%) were adults and 2229 (47%) were children. Those < 18 years included 549 (12%) aged 1 to 4 years, 750 (16%) aged 5 to 11 years, and 930 (19%) aged 12–17 years.

**Table 2:** Baseline characteristics of the participants included in the PREVAC trial

	Version 2.0		Version 3.0		Version 4.0		All versions	
	No.	Pct.	No.	Pct.	No.	Pct.	No.	Pct.
**Country**								
Guinea	263	49.0	1068	73.7	999	35.7	2330	48.7
Liberia	274	51.0	382	26.3	477	17.0	1133	23.7
Sierra Leone	0	0.0	0	0.0	708	25.3	708	14.8
Mali	0	0.0	0	0.0	618	22.1	618	12.9
**Gender**								
Male	333	62.0	743	51.2	1536	54.8	2612	54.5
Female	204	38.0	707	48.8	1266	45.2	2177	45.5
**Age**								
1–4 years	0	0.0	82	5.7	467	16.7	549	11.5
5–11 years	0	0.0	283	19.5	467	16.7	750	15.7
12–17 years	41	7.6	422	29.1	467	16.7	930	19.4
18–34 years	354	65.9	413	28.5	946	33.8	1713	35.8
35–54 years	114	21.2	187	12.9	368	13.1	669	14.0
≥ 55 years	28	5.2	63	4.3	87	3.1	178	3.7
Median (25th, 75th %)	26 (20–35)		17 (11–28)		18 (8–27)		18 (11–28)	
**Indicators of increased risk**								
Contact with case	4	0.7	7	0.5	2	0.1	13	0.3
Work entailing contact	3	0.6	1	0.1	1	0.1	5	0.1
**Clinical examination** Median (25th, 75th %)								
Weight (kg)	64 (57–71)		54 (33–65)		52 (25–63)		54 (31–65)	
Height (cm)	167 (160–173)		159 (143–168)		158 (128–168)		160 (140–169)	
BMI*	22.4 (20.4–25.4)		20.2 (17.1–23.6)		20.5 (17.2–23.6)		20.7 (17.6–23.8)	
Arm circumference** (cm)			15.8 (15.0–16.4)		15.5 (14.6–16.2)		15.5 (14.6–16.2)	
Body temperature (^o^ C)	36.5 (36.4–36.7)		36.5 (36.4–36.7)		36.6 (36.4–36.8)		36.6 (36.4–36.8)	
**Infection test results**								
HIV positive	14	2.6	15	1.0	25	0.9	54	1.1
Syphilis positive	11	2.0	16	1.1	18	0.6	45	0.9
**No. enrolled**	**537**		**1450**		**2802**		**4789**	

*Age 6 years or older

**Age 5 years or younger

At baseline, 13 (0.3%) participants reported having contact with someone with Ebola either recently or in the past, while 5 (0.1%) participants reported that their recent or past work involved contact with a living or dead person with Ebola or with the bodily fluid from a patient.

Among participants enrolled, the median weight at baseline was 54 kg [IQR 31–65], while the median body mass index (BMI) among participants aged ≥ 6 years was 20.7 [17.6–23.8] at baseline. Among children aged ≤ 5 years, the median mid upper arm circumference was 15 cm [36.4–36.8].

### Baseline laboratory measurements

Biochemical and hematological baseline laboratory measurements are presented pooled for versions 2.0, 3.0, and 4.0 in Tables 3 and 4 for females and males respectively. Laboratory measurements are detailed by country in Additional file 1: Appendix – Table 1.

**Table 3:** Baseline laboratory measurements of female participants included in the PREVAC trial (pooled for versions 2.0, 3.0, and 4.0)

Baseline laboratory measurements: females								
	Age 18+ (***n*** = 1118 )		Age 12–17 (***n*** = 429)		Age 5–11 (***n*** = 353)		Age 1–4 (***n*** = 276 )	
	Mean	SD	Mean	SD	Mean	SD	Mean	SD
**Biochemistry**								
ALT (U/L)	10.9	10.6	9.3	7.3	11.7	11.8	13.8	38.2
AST (U/L)	14.2	9.6	14.9	6.7	20.7	11.6	28.9	30.6
Creatinine (mg/dl)	0.80	0.15	0.66	0.15	0.53	0.13	0.38	0.10
Potassium (mmol/L)	4.1	0.4	4.2	0.3	4.1	0.4	4.3	0.5
**Hematology**								
White blood cells (x10³/μL)	6.1	2.1	6.2	1.8	7.0	1.9	8.9	2.7
Neutrophils (x10³/μL)	2.9	1.2	2.7	1.1	2.9	1.4	3.2	1.4
Lymphocytes (x10³/μL)	2.5	1.4	2.6	0.9	3.0	0.9	4.5	1.7
Eosinophils (x10³/μL)	0.21	0.26	0.26	0.37	0.35	0.43	0.39	0.64
Monocytes (x10³/μL)	0.46	0.18	0.52	0.20	0.60	0.22	0.77	0.34
Basophils (x10³/μL)	0.08	0.10	0.08	0.04	0.10	0.05	0.15	0.09
Hemoglobin (g/dl)	12.3	1.3	12.3	1.3	11.9	1.2	10.7	1.2
Hematocrit (%)	37.5	3.6	37.2	3.5	36.0	3.4	33.2	3.2
Platelets (x10³/μL)	264.2	71.7	288.4	76.4	321.4	87.2	394.3	111.0
Red blood cells (x10^6^/μL)	4.5	0.5	4.6	0.5	4.6	0.5	4.6	0.5
Red cell distribution width (%)	13.3	1.6	13.3	1.6	13.6	1.6	14.9	2.3
Mean corpuscular volume (fL)	83.8	7.0	80.9	7.3	78.1	6.4	72.8	8.0

Lower limit of detection is imputed for undetectable results

**Table 4:** Baseline laboratory measurements of male participants included in the PREVAC trial (pooled for versions 2.0, 3.0, and 4.0)

Baseline laboratory measurements: males								
	Age 18+ (***n*** = 1440 )		Age 12–17 (***n*** = 501 )		Age 5–11 (***n*** = 396)		Age 1–4 (***n*** = 273)	
	Mean	SD	Mean	SD	Mean	SD	Mean	SD
**Biochemistry**								
ALT (U/L)	15.5	28.9	13.8	22.3	12.8	14.5	12.3	16.0
AST (U/L)	19.8	22.3	20.9	17.0	21.9	11.6	30.2	27.3
Creatinine (mg/dl)	1.00	0.21	0.75	0.22	0.53	0.14	0.39	0.10
Potassium (mmol/L)	4.2	0.4	4.3	0.4	4.2	0.4	4.3	0.5
**Hematology**								
White blood cells (x10³/μL)	5.4	1.5	5.9	1.6	7.3	3.4	9.0	3.0
Neutrophils (x10³/μL)	2.4	1.1	2.4	1.1	2.9	1.3	3.0	1.3
Lymphocytes (x10³/μL)	2.2	0.7	2.5	0.7	3.2	2.5	4.6	2.1
Eosinophils (x10³/μL)	0.30	0.55	0.39	0.50	0.54	0.81	0.43	0.53
Monocytes (x10³/μL)	0.44	0.16	0.53	0.20	0.60	0.25	0.82	0.33
Basophils (x10³/μL)	0.08	0.03	0.09	0.04	0.11	0.13	0.15	0.08
Hemoglobin (g/dl)	14.2	1.5	12.9	1.5	11.6	1.2	10.4	1.3
Hematocrit (%)	43.1	4.3	39.0	4.2	35.0	3.5	32.1	3.6
Platelets (x10³/μL)	233.1	67.7	270.6	79.3	309.2	89.6	409.5	139.6
Red blood cells (x10^6^/μL)	5.1	0.6	4.9	0.5	4.5	0.5	4.5	0.6
Red cell distribution width (%)	13.1	1.4	13.4	1.6	13.7	1.5	15.4	2.1
Mean corpuscular volume (fL)	84.8	6.7	80.4	6.3	77.2	6.0	71.5	7.4

Lower limit of detection is imputed for undetectable results

Among 2560 adult participants who had an HIV test, 54 (2.1%) tested positive for HIV. All children enrolled in the study were HIV negative at baseline. Both adults and children were tested for syphilis at baseline and 45 (0.9%) participants tested positive and were referred for treatment (Table 2).

### Preliminary data on antibody titers at baseline

Preliminary baseline antibody data were available for 23% (1090) of the study population. This sample represented the samples transferred to and processed at the LIBR in Liberia before September 23, 2019. On the 1090 participants, 676 (62%) were from the Mambolo site, 414 (38%) were from Landreah.

At baseline, the median IgG antibody level against EBOV was 72 enzyme-linked immunosorbent assay units (EU) per milliliter (IQR, 50 to 116; 5th and 95th percentiles, 33 to 330). A total of 2.4% of participants had antibody titers of greater than 607 EU/mL; 11.7% had titers greater than or equal to 200 EU/mL (Table 5). These preliminary results indicate that antibody titers prior to vaccination are similar for males (84.2 EU/mL) and females (79.8) and may be higher in adolescent (86.9 EU/mL) and adults (83.7 EU/mL) compared to young children (68.2 EU/mL in 1–4 years old and 78.4 in 5–11 years old) (Additional file 1: Appendix 8).

**Table 5:** Preliminary baseline antibody data (based on a subset of samples of the PREVAC trial participants from version 2.0, 3.0 and 4.0 that were not randomly selected)

PREVAC baseline antibody titers by age						
Age	No.	GMT* EU/mL	Median (IQR) EU/mL	5th, 95th percentile	≥ 200 EU/mL No. (%)	> 607 EU/mL No. (%)
**All children**	413	81.3	68 (47, 116)	30, 451	54 (13.1%)	15 (3.6%)
1–4 years	44	68.2	56 (43, 90)	26, 227	4 (9.1%)	2 (4.5%)
5–11 years	162	78.4	68 (46, 119)	28, 351	21 (13.0%)	4 (2.5%)
12–17 years	207	86.9	71 (49, 117)	34, 470	29 (14.0%)	9 (4.3%)
**All adults**	677	83.7	74 (52, 114)	34, 317	74 (10.9%)	11 (1.6%)
18–25 years	392	86.0	75 (52, 122)	33, 335	45 (11.5%)	8 (2.0%)
26–45 years	205	78.7	69 (50, 103)	35, 269	19 (9.3%)	2 (1.0%)
46+ years	80	86.5	73 (54, 126)	37, 300	10 (12.5%)	1 (1.3%)
**All ages**	1090	82.8	72 (50, 116)	33, 330	128 (11.7%)	26 (2.4%)

*Geometric mean titer of antibody testing through 22 September 2019

## Discussion

The successful planning and conduct of this randomized trial of three Ebola vaccine strategies involving six sites within four West African Countries, three sponsors, one NGO, and two pharmaceutical companies is a milestone in collaborative Ebola vaccine research. The results of PREVAC will inform safety and characterize both short-term and long-term immune responses for three vaccine strategies for the prevention of Ebola virus disease in adults and children.

Prior to the design and conduct of PREVAC, most Ebola vaccine phase II trials did not involve children [12]. The involvement of children aged 1–17 in PREVAC is key in evaluating the safety profile of the different candidate vaccines in children. Almost half (2229 out of 4789) of participants who enrolled in PREVAC were children aged 1–17 years. Considering that the percentage of children who were infected with EVD during the 2014–2015 epidemic in West Africa was 16% and these children had a higher case fatality rate than adults [13], the results from this trial will provide important information to guide the vaccination of children in future epidemics such as the one ongoing in DRC where children are being vaccinated.

Whether the different vaccination approaches are able to confer durable antibody responses after vaccination remains an important question and currently limited data exist to address this question. The PREVAC and PREVAC-UP trials will evaluate the durability of immune responses with follow-up of the participants up to 60 months after vaccination. These data will provide important information when considering a preventive vaccination strategy for at-risk populations, including health-care and front-line workers who may face ongoing exposure to cases over repeated outbreaks.

The use of a placebo in a clinical trial of a vaccine considered to be efficacious in preventing EVD would be difficult to justify during an active outbreak. However, its use was considered ethical in the current non-epidemic context in the four countries participating in the trial. Placebo controls are an important strength of this trial for the evaluation of safety outcomes. A provision was included in the design of PREVAC that indicated that the use of placebos and the study design would be reconsidered if Ebola returned to these countries during the trial. With the planned long-term follow-up, the same provision will apply. Given the recent licensure of the rVSV∆G-ZEBOV-GP by both the EMA and the FDA, it would be unethical to conduct an efficacy trial with a placebo arm.

The inclusion of the two most promising vaccines was also a strength of the trial. The opportunity to collaborate with the respective companies producing the vaccine and the use of pooled placebo arms were efficient and appropriate given the urgency of the public health need.

There were efforts to achieve equal demographic distribution among participants. An enrollment monitoring team was established to ensure that there was diversity in the ages of children enrolled, especially during enrollment into version 4.0 of the trial. This system allowed a good representation of each age cohort in the trial as was done in PREVAIL I [7].

The design of the trial had to evolve quickly over time to adapt to emerging safety information and the realities of vaccine production. The PREVAC trial began enrollment with version 2.0 with only two arms and one vaccine strategy while awaiting additional safety investigation on a Merck vaccine lot and the availability of a new lot of the rVSV∆G-ZEBOV-GP vaccine. The 5-arm design was split into two successive phases, version 3.0 (diluted rVSV∆G-ZEBOV-GP) and version 4.0 (undiluted rVSV∆G-ZEBOV-GP), because the lot release potency of rVSV∆G-ZEBOV-GP vaccine was 9.4 × 10^7^ pfu/mL, which, while within the pre-specified release range of the manufacturer, was greater than the dose with which the investigators had experience in Liberia in PREVAIL I [7]. Variation in the potency in live virus vaccines is common. Vaccine manufacture and release for potency is based upon defined specifications and always encompasses a range with a lower limit. The lower limit is determined during development and is defined by the lowest dose for which there is demonstrated efficacy. The lower limit for potency must still be valid at the end of shelf-life in order to ensure that the vaccine is still efficacious until its defined expiry. Given this, the initial potency of any given lot is typically considerably higher than the nominal dose.

Prior to the conduct of PREVAC, only half the trial sites had prior experience on mobilization and community awareness programs for enrollment and retention of participants in clinical trials. Community engagement is pivotal to recruitment and retention of participants in clinical trials [14, 15]. While establishing clinical trial capacity in new sites and upgrading existing sites, community awareness was an integral component that led to achievement of a high rate of enrollment of adults and children in a short period of time and a very low drop-out rate. At most sites, the number of eligible volunteers willing to enroll exceeded the number of participants required. Based on our experience, the development of plans for engaging the community will be an important consideration in future studies carried out by our partnership.

The implementation of a clinical trial such as PREVAC builds and strengthens capacity in clinical research for the personnel involved and physical infrastructure at each site. These personnel have also been able to bring their expertise to other African countries, for example some of site staff went to DRC to strengthen the Ebola clinical research capacity deployed to fight the current epidemic. Such trials can also lead to better training programs for clinical researchers in African institutions.

In conclusion, the results of the PREVAC trial will extend our knowledge of the safety and long-term immunogenicity of the two most promising vaccines to prevent Ebola.

## Trial status

The recruitment of participants for this trial has been finished, and the 1-year follow-up (primary outcome) of the trial was completed by December 24, 2019. The 5-year follow-up is planned to be completed in December 2023. The current version of the protocol is version 5.0 (October 3, 2019). This manuscript describing the study protocol was submitted after all participants were included in the different versions of the protocol. This was done in order to be able to present all the versions and to present the baseline characteristics of the participants included in the PREVAC study.

## Supplementary Information

**Additional file 1: Appendix 1.** Secondary objectives. **Appendix 2.** Sample size considerations and statistical analysis. **Appendix 3.** PREVAC study team. **Appendix 4.** Trial organization. **Appendix 5.** Assent and consent forms. **Appendix 6.** Participants in the PREVAC trial. **Appendix 7.** Enrollment of the participants in the PREVAC trial. **Appendix 8.** Baseline antibody data.

## Data Availability

The datasets generated and/or analyzed during the current study are not publicly available but are available on reasonable request addressed to the corresponding author.
