# Correction to: Partnership for Research on Ebola VACcination (PREVAC): protocol of a randomized, double-blind, placebo-controlled phase 2 clinical trial evaluating three vaccine strategies against Ebola in healthy volunteers in four West African countries

**DOI:** 10.1186/s13063-021-05572-3

**Published:** 2021-09-01

**Authors:** Moses Badio, Edouard Lhomme, Mark Kieh, Abdoul Habib Beavogui, Stephen B. Kennedy, Seydou Doumbia, Bailah Leigh, Samba O. Sow, Alpha Diallo, Daniela Fusco, Matthew Kirchoff, Monique Termote, Renaud Vatrinet, Deborah Wentworth, Helène Esperou, H. Clifford Lane, Jerome Pierson, Deborah Watson-Jones, Céline Roy, Eric D’Ortenzio, Brian Greenwood, Genevieve Chêne, Laura Richer, James D. Neaton, Yazdan Yazdanpanah, the PREVAC study team

**Affiliations:** 1 Partnership for Research on Ebola Virus in Liberia (PREVAIL), Monrovia, Liberia; 2 Univ. Bordeaux, Inserm, Bordeaux Population Health Research, Center, UMR 1219, CHU Bordeaux, CIC 1401, EUCLID/F-CRIN Clinical Trials Platform, F-33000 Bordeaux, France; 3 Centre National de Formation et de Recherche en Santé Rurale de Mafèrinyah, Mafèrinyah, Guinea; 4 University of Sciences, Technique and Technology of Bamako, Bamako, Mali; 5 College of Medicine and Allied Health Sciences (COMAHS), University of Sierra Leone, Freetown, Sierra Leone; 6 Centre pour le Développement des Vaccins, Bamako, Mali; 7 INSERM, Pôle de Recherche Clinique, 75013 Paris, France; 8 National Institute of Allergy and Infectious Diseases, Bethesda, MD USA; 9 REACTing, Institut Thématique Immunologie, Inflammation, Infectiologie et Microbiologie, Inserm, Paris, France; 10 Division of Biostatistics, School of Public Health, University of Minnesota, Minneapolis, MN USA; 11 London School of Hygiene & Tropical Medicine, London, UK; 12 AP-HP, Hôpital Bichat-Claude Bernard, Service de Maladies Infectieuses et Tropicales, F-75018 Paris, France

**Correction to: Trials 22, 86 (2021)**

**https://doi.org/10.1186/s13063-021-05035-9**

Following the publication of the original article [1], we were notified of an error in the affiliation of 3 authors of the article: Celine Roy, Laura Richert and Genevieve Chene.

Their affiliation was initially mentioned as: “Partnership for Research on Ebola Virus in Liberia (PREVAIL), Monrovia, Liberia”

However, their correct affiliation is: Univ. Bordeaux, Inserm, Bordeaux Population Health Research Center, UMR 1219, CHU Bordeaux, CIC 1401, EUCLID/F-CRIN Clinical Trials Platform, F-33000, Bordeaux, France.tp
