# Supplementary material for: Partnership for Research on Ebola VACcination (PREVAC): protocol of a randomized, double-blind, placebo-controlled phase 2 clinical trial evaluating three vaccine strategies against Ebola in healthy volunteers in four West African countries
Source: Trials. 2021 Jan 23;22:86. doi: 10.1186/s13063-021-05035-9 (PMC7823170; doi:10.1186/s13063-021-05035-9)
Supplement: Supplementary file 1 — Additional file 1: Appendix 1. Secondary objectives. Appendix 2. Sample size considerations and statistical analysis. Appendix 3. PREVAC study team. Appendix 4. Trial organization. Appendix 5. Assent and consent forms. Appendix 6. Participants in the PREVAC trial. Appendix 7. Enrollment of the participants in the PREVAC trial. Appendix 8. Baseline antibody data. [file 13063_2021_5035_MOESM1_ESM.docx]

**APPENDIX**

**Appendix 1. Secondary objectives**

The primary objective for each company is shown below.

In order to facilitate Merck regulatory filings and bridging of immune responses of the rVSV∆G-ZEBOV-GP vaccine from this study to other studies and between pediatric and adult populations, the following objective will be assessed specifically for those in the two rVSV∆G-ZEBOV-GP vaccine groups separately for adults and children:

- To compare the rVSV∆G-ZEBOV-GP vaccine (pooled rVSV∆G-ZEBOV-GP groups) with the matched placebo group for antibody response 28 days after randomization (prime vaccination).

In order to facilitate Janssen regulatory filings of the Ad26/MVA vaccine, the following objective will be assessed specifically for those in the Ad26/MVA vaccine group separately for adults and children:

- To compare the Ad26.ZEBOV,MVA-BN-Filo vaccine group with the matched placebo group for antibody response 3 months after randomization (approximately 28 days after the second dose vaccination).

The following secondary objectives will be addressed. Unless otherwise stated, the objectives will be addressed separately for adults and children:

- To compare the groups given the Ad26.ZEBOV prime vaccine and the rVSV∆G-ZEBOV-GP prime vaccine (both rVSV∆G-ZEBOV-GP groups, with and without the boost, combined) each with the placebo group for the antibody response 14 days after randomization (immediacy of response).
- To compare the groups given the Ad26.ZEBOV prime vaccine and the rVSVΔG-ZEBOV-GP prime vaccine (both rVSVΔG-ZEBOV-GP groups combined) each with the pooled placebo group for the antibody response 14 days after randomization (prime vaccination) (immediacy of response).
- To compare each of the vaccine groups versus the pooled placebo group for the antibody response profile using measurements at 7, 14, 28, 56, 63 days and at 3, 6 and 12 months after randomization.
- To compare each of the vaccine groups with the pooled placebo group for the incidence of SAEs during 12 months of follow-up.
- To compare the groups given the Ad26.ZEBOV prime vaccine and the rVSVΔG-ZEBOV-GP prime vaccine (both rVSVΔG-ZEBOV-GP groups combined) each with the pooled placebo group for the percent with injection site reactions and AEs graded for severity, including targeted symptoms, during the first week following randomization (including the daily contacts for children only).
- To compare the groups given the Ad26.ZEBOV prime vaccine and the rVSVΔG-ZEBOV-GP prime vaccine (both rVSVΔG-ZEBOV-GP groups combined) each with the pooled placebo group for percent reporting injection site reactions and AEs graded for severity, including targeted symptoms, following prime vaccination at the vaccination visit, and through 7, 14, and 28 days after the prime vaccination.
- To compare each of the vaccine groups with the pooled placebo group for changes from baseline in biochemical markers and complete blood count (CBC) measurements at 7 days after randomization (children only).
- To compare the Ad26.ZEBOV,MVA-BN-Filo and rVSVΔG-ZEBOV-GP boost strategies with the pooled placebo group for changes from baseline in biochemical markers and blood count measurements at 63 days after randomization (children only).
- To compare the Ad26.ZEBOV,MVA-BN-Filo and rVSVΔG-ZEBOV-GP boost strategies with the pooled placebo for percent with injection site reactions and AEs graded for severity, including targeted symptoms, immediately following the booster vaccination until approximately 35 days after the booster vaccination.
- To compare the three vaccine strategies versus the pooled placebo group for long-term antibody response at 24, 36, 48 and 60 months following randomization.
- To compare long-term safety at month 24, 36, 48 and 60 following the three vaccine strategies with the pooled placebo group.
- To compare antibody responses and safety outcomes of each of the vaccination strategies versus the pooled placebo group in subgroups defined by age, gender, country, whether the volunteer was a close contact of an Ebola case, had laboratory abnormalities at baseline, or had specific co-morbidities (in particular HIV and nutritional status as measured by body mass index).
- For adults and children combined, to compare antibody responses and safety outcomes for each of the vaccination strategies versus placebo.
- To carry out operational research which includes ethnographic, participatory and/or qualitative (i.e., focus groups and individual interviews) studies to: 1) identify issues relevant to understanding the acceptability of the trial, the social issues surrounding informed consent, with the primary goal of informing efforts to ensure autonomous fully informed individual consent and assent for minors; 2) describe participants' and caregivers' experience in the trial, and identify barriers and develop solutions to support trial adherence in a culturally sensitive and ethically appropriate way; and 3) understand prevailing representations and affects surrounding the epidemic (including rumors), Ebola and other vaccines, the trial and other relevant phenomena in order to ensure effective communication around the trial.
- In a subsample of adults, T cell and memory B cell responses for the three vaccine strategies versus placebo will be compared.
- In a subsample of children, to compare the rVSVΔG-ZEBOV-GP vaccine strategies with the pooled placebo group for shedding of rVSV-ZEBOV-GP RNA.
- In subsample, to examine the impact of malaria on persistence of immune responses.
- To evaluate AEs of lesser severity, including injection site reactions and targeted symptoms, following the initial vaccination and following the booster.

**Appendix 2. Sample size considerations and statistical analysis**

Analyses will be carried out according to a statistical analysis plan that is updated prior to unblinding. The primary analysis will be based on participants enrolled under Version 4.0. Separate analyses will be carried out for participants enrolled under Version 2.0 and 3.0. Some of the analyses will pool data across all protocol versions and these are described in the statistical analysis plan. Adults and children will be analyzed separately and combined. Baseline characteristics and follow-up completeness will be summarized by vaccine group. Participants with serological positivity for Ebola at baseline will be included in the main analysis. A robustness analysis will be performed excluding these participants.

Prior to carrying out the endpoint analyses which pool the two placebo groups, the two placebo groups will be compared for the immunogenicity and safety outcomes. The primary analyses for adult and children will be carried out at the 0.0167 (2-sided) level of significance and, as currently planned, will compare the three vaccine strategies with the pooled placebo group for the percent with a positive antibody response at 12 months (defined as increase in the log_10_ titer by a factor of 4 or more among those with antibody titers < 608 EU/mL at entry). Analyses will also be carried out for randomized participants irrespective of the antibody level at entry.

Each pair-wise comparison will be carried out using Mantel-Haenszel chi-square statistics which consider stratification by vaccination center. Similar methods will also be used to compare groups for the immediacy of the antibody response at 14 days and at other, including 28 days after randomization and 28 days following the booster vaccination. The two additional primary objectives for immune responses, pooled rVSVΔG-ZEBOV-GP groups versus pooled placebo groups 28 days after randomization for Merck, and Ad26.ZEBOV,MVA-BN-Filo versus pooled placebo groups approximately 28 days following booster vaccination for Janssen, will be carried out the 0.05 (2-sided) level of significance.

All comparisons (for adults, children, and for adults and children combined) of immune responses and safety outcomes at other time points will be carried out at the 0.0167 (2-sided) level of significance when there are three pair-wise comparisons made (e.g., each vaccine strategy versus pooled placebo) and at the 0.025 (2-sided) level of significance when there are two pair-wise comparisons made (e.g., for comparisons at days 7, 14, 28 and 56 with the pooled placebo group in which the two rVSVΔG-ZEBOV-GP strategies are pooled).

SAEs will be classified by system organ class according to MedDRA®. The total number of events and the number of participants with at least one event will be reported. Mantel-Haenszel chi-square statistics will be used for each pair-wise comparison of the number of participants with an SAE through 12 months of follow-up; time-to-event methods, e.g., Kaplan-Meier, Cox regression, will also be used to compare groups for SAEs over the 12 month follow-up period. Analyses will also be carried out for SAEs judged to be related to the vaccine. Similar analyses will be performed for SAEs reported through 60 months.

Each vaccine group will be compared with the pooled placebo group using Mantel-Haenszel chi-square tests and time-to-event methods for diagnoses of laboratory-confirmed malaria through 12 months of follow-up.

For safety and efficacy outcomes assessed before the booster at 56 days, the two groups given the rVSVΔG-ZEBOV-GP prime vaccination will be combined for comparisons with pooled placebo group. For outcomes that are assessed after 56 days, each of the three vaccination strategies will be compared with the pooled placebo group.

Mantel-Haenszel chi-square tests will be used to compare each vaccine with placebo for the percent with grade 3 or 4 AEs and with targeted symptoms reported at 7, 14, and 63 days. Similar methods will be used to compare injection site reactions reported immediately following prime vaccination and at 7 and 14 days. Likewise, treatment differences in injection site reactions reported immediately following booster vaccination and through day 63 (7 days after booster vaccination) will be summarized with similar methods. In the event that there is evidence that safety outcomes differ between the two placebo groups, analyses will also be performed for each vaccine versus its matched placebo.

Longitudinal mixed models will be used to compare log-transformed antibody titers collected at multiple follow-up time points during follow-up. Results will be summarized as geometric means and geometric mean ratios. Baseline titer level will be included as a covariate. These analyses will be supplemented with scatter plots that display fold increases in antibody titers, line plots, box plots and reverse cumulative distributions of antibody levels at each visit for each vaccine group.

For children, changes in biochemical and CBC test values after 7 and 63 days will be compared for each vaccine versus the pooled placebo group using analysis of covariance with the baseline laboratory value as a covariate. Laboratory test results will be graded for severity according to the Division of AIDS (DAIDS) AE Table and local laboratory norms, and the proportion with grade 3 or 4 laboratory abnormalities will be compared. For children, findings based on contacts during the 7 days following the prime and booster vaccination will be summarized each day. Trends over the 7 days will be examined for each vaccine versus placebo comparison.

Subgroup analyses for the primary endpoints and major secondary outcomes, including SAEs and other safety data, will be performed to determine whether vaccine versus placebo differences vary qualitatively across various baseline-defined subgroups. Subgroup analyses will be performed using regression analysis by age, gender, country, vaccination center, health care worker or job involving close contact with EVD cases, baseline laboratory abnormalities, HIV status, and nutritional status as determined by body mass index. An overall test of heterogeneity will provide evidence of whether the magnitude of the vaccine pair-wise comparison for efficacy/safety outcomes varies across these baseline subgroups.

In addition to the separate analysis for adults and children for each vaccine group versus placebo for antibody levels and safety outcomes, the endpoints will also be summarized pooling the data for adults and children. Methods similar to those described above will be used after stratification on age.

**Appendix 3. PREVAC study team**

1. **Executive Commitee**

The Executive Committee is composed of representatives of the sponsors (Yves Lévy, Cliff Lane, Peter Piot), and the coordinating investigator (Yazdan Yazdanpanah).

1. **Trial Steering Committee (TSC)**

Voting Members: Abdoul Habib Beavogui, Geneviève Chêne, Seydou Doumbia, Hélène Espérou, Brian Greenwood, Stephen Kennedy, Cliff Lane, Bailah Leigh, Yves Lévy, James Neaton, Jerome Pierson, Peter Piot, Samba Sow, Deborah Watson-Jones, Yazdan Yazdanpanah (Chair).

Non-Voting Members: Augustin Augier, Beth-Ann Coller, Sandrine Couffin-Cardiergues, Alpha Diallo, Christine Lacabaratz, Maarten Leyssen.

Permanent Observers: Boni Ale, Moses Badio, Eric Barte de Saint Fare, Cécilia Campion, Siew Pin Chai, Laurie Connor, Mahamadou Diakite, Eric D'Ortenzio, Moussa Moise Doumbia, Suzanne Fleck, Birgit Grund, Oumar Guindo, David Ishola, Mark Kieh, Daniela Manno, Kim Offergeld, Cynthia Osborne, Sushma Patel, Stephany Pong, Laura Richert, Cynthia Robinson, Céline Roy, Christine Schwimmer, Jakub Simon, Mili Tapia, Renaud Vatrinet, Deborah Wentworth, Jimmy Whitworth, Aurelie Wiedemann.

1. **Independent Data and Safety Monitoring Board (DSMB)**

Lisa Cooper (chair), Salim Abdulla, David DeMets, Albert Faye, Scott Hammer, Amadou Traoré, Ann Sarah Walker

1. **PREVAC Study Team (TSC, TMT and WG members)**

Coulibaly Abdoulaye^20^, Jamilia Aboulhab^2^, Pauline Akoo^3^, Esther Akpa^2^, Robert Akpata^1^, Sara Albert^18^, Boni Maxime Ale^4^, Benetta C. Andrews^10^, Stephane Anoma^6^, Saw-San Assiandi^1^, Augustin Augier^6^, Ken Awuondo^3^, Moses Badio^10^, Aminata Bagayoko^1^, Nyasha Bakare^14^, Abby Balde^18^, Lamin Molecule Bangura^3^, Kesha Barrington^18^, Eric Barte de Saint Fare^6^, Beth Baseler^18^, Ali Bauder^13^, Claire Bauduin^4^, Luke Bawo^10^, Abdoul Habib Beavogui^9^, Michael Belson^2^, Marion Bererd^6^, Teedoh Beyslow^10^, Blandine Binachon^4^, Julie Blie^10^, Viki Bockstal^14^, Youba Boire^21^, Patricia Boison^18^, Fatorma Bolay^22^, Aliou Boly^6^, Anne Gael Borg^6^, Donna Bowers^3^, Sarah Browne^10^, Barbara Cagniard^1^, Kelly Cahill^2^, Aissata Abdoulaye Camara^6^, Keira Camara^1^, Modet Camara^6^, Cécilia Campion^4^, Jennifer Cash^2^, Siew Pin Chai^14^, Francois Chambelin^1^, Keita Chieck^6^, Geneviève Chêne^4^, Séverine Ciancia^1^, Papa Ndiaga Cisse^15^, Elfrida Clide^18^, Céline Colin^4^, Beth-Ann Coller^13^, Djélikan Siaka Conde^1^, Katherine Cone^2^, Laurie Connor^13^, Nicholas Connor^3^, Joseph Boye Cooper^10^, Sandrine Couffin-Cardiergues^1^, Fatoumata Coulibaly^1^, Mariam Coulibaly^20^, Sandrine Dabakuyo-Yonli^4^, Djeneba Dabitao^21^, Thierry Damerval^1^, Bionca Davis^5^, Gibrilla Fadlu Deen^11^, Eline Dekeyster^14^, Jean-François Delfraissy^1^, Christelle Delmas^1^, Rokia Dembele^20^, Mahamadou Diakite^21^, Alpha Diallo^1^, Mamadou Saliou Diallo^6^, Ayouba Diarra^21^, Oualy Diawara^20^, Bonnie Dighero-kemp^2^, Samba Diop^22^, Waly Diouf^15^, Laurie Doepel^2^, Eric D'Ortenzio^1,7,8^, Seydou Doumbia^12^, Moussa Moise Doumbia^20^, Macaya Douoguih^14^, Alain DuChêne^5^, Michael Duvenhage^18^, Risa Eckes^2^, Avril Egan^14^, Luisa Enria^3^, Hélène Espérou^1^, Cécile Etienne^1^, Allison Eyler^18^, Sylvain Faye^15^, José Fernandez^1^, Suzanne Fleck^3^, Vemy Fofana^6^, Kokulo Franklin^10^, Daniela Fusco^1^, Auguste Gaddah^14^, Marylène Gaignet^1^, Katherine Gallagher^3^, Julia Garcia Gozalbes^1^, Greg Grandits^5^, Maima Gray^10^, Brian Greenwood^3^, Astrid Greijer^14^, Louis Grue^18^, Birgit Grund^5^, Oumar Guindo^21^, Swati Gupta^13^, Fadima Haidara^20^, Benjamin Hamze^1^, Emma Hancox^3^, Gavin Hart^14^, Jean-Christophe Hébert^1^, Esther Heijnen^14^, Patricia Hensley^3^, Lisa Hensley^2^, Elisabeth Higgs^2^, Trudi Hilton^3^, Preston Holley^18^, Marie Hoover^17^, Natasha Howard^3^, Melissa Hughes^13^, Dicko Ilo^21^, Jen Imes^18^, Skip Irvine^13^, David Ishola^3^, Will Jacob^2^, Yvonne Jato^2^, Melvin Johnson^10^, Morrison Jusu^3^, Aboubacar Sidiki Kaba^6^, Myriam Kante^4^, Judith Katoudi^6^, Sakoba Keita^16^, Stephen Kennedy^10^, Babajide Jide Keshinro^14^, Brian Khon^3^, Hassan Kiawu^10^, Mark Kieh^10^, Matt Kirchoff^2^, Mamoudou Kodio^20^, Lamine Koivogui^24^, Tania Kombi^6^, Stacy Kopka^18^, Dickens Kowuors^3^, Christine Lacabaratz^1^, Boris Lacarra^1^, Laurie Lambert^18^, Cliff Lane^2^, Shona Lee^3^, Shelley Lees^3^, Annabelle Lefevre^1^, Bailah Leigh^11^, Frederic Lemarcis^1^, Yves Lévy^1^, Claire Levy-Marchal^1^, Jemilla Lewally^3^, Maarten Leyssen^14^, Edouard Lhomme^4^, Ken Liu^13^, Brett Lowe^3^, Julia Lysander^10^, Claire Madelaine^1^, Ibrah Mahamadou^6^, Daniela Manno^3^, Johnathan Marchand^19^, Siegfried Marynissen^14^, Moses B.F. Massaquoi^10^, Laure Masson^1^, Charly Matard^4^, Onorato Matthew^13^, John McCullough^17^, Noemie Mercier^1^, Pauline Michavila^6^, Tracey Miller^18^, Alejandra Miranda^18^, Soumaya Mohamed^6^, Tom Mooney^3^, Hans Morsch^3^, Dally Muamba^6^, Rita Lukoo Ndamenyaa^6^, James Neaton^5^, Désiré Neboua^1^, Micki Nelson^13^, Kevin Newell^18^, Vinh-kim Nguyen^25^, Leslie Nielsen^18^, Millimouno Niouma^6^, Kim Offergeld^14^, Matthew Onorato^13^, Uma Onwuchekwa^23^, Susan Orsega^2^, Inmaculada Ortega-Perez^1,8^, Cynthia Osborne^18^, Tuda Otieno^3^, Sushma Patel^13^, Nathan Peiffer-Smadja^1^, Robert Phillips^3^, Jerome Pierson^2^, Peter Piot^3^, Micheal Piziali^2^, Stephany Pong^1^, Calvin Proffitt^18^, Alexandre Quach^1^, Corina Ramers-verhoeven^14^, Nadeeka Randunu^18^, Laura Richert^4^, Priscille Rivière^1^, Cynthia Robinson^14^, Griet Van Roey^14^, Céline Roy^4^, Amy Falk Russell^13^, Mohamed Samai, Sibiry Samake^21^, Ballan Sangare^20^, Ibrahim Sanogo^21^, Yeya Sadio Sarro^21^, Lorraine Sautter^1^, Mélanie Saville^14^, Serge Sawadogo^6^, Maxime Schvartz^1^, Christine Schwimmer^4^, Fatou Secka^3^, Jacques Seraphin^6^, Denise Shelley^18^, Sophia Siddiqui^2^, Jakub Simon^13^, Shelly Simpson^18^, Billy Muyisa Sivahera^6^, Irvine Skip^13^, Karen Slater^2^, Mary Smolskis^2^, Elizabeth Smout^3^, Emily Snowden^3^, Anne-Aygline Soutthiphong^4^, Samba Sow^23^, Ydrissa Sow^2^, Daniel Splinter^14^, Simone Spreng^14^, Helen Stapleton^14^, Jeroen Stoop^14^, Mary Sweeney^14^, Sienneh Tamba^10^, Mili Tapia^23^, Jemee Tegli^10^, Monique Termote^4^, Rodolphe Thiebaut^4^, Greg Thompson^5^, John Tierney^2^, Abdoulaye Touré^24^, Stacey Traina^13^, Awa Traore^20^, Moussa Traore^20^, Tijili Tyee^10^, David Vallée^1^, Katrien Van Der Donck^14^, Renaud Vatrinet^1^, Nadia Verbruggen^14^, Corine Vincent^4^, Susan Vogel^2^, Cedrick Wallet^4^, Deborah Watson-Jones^3^, Deborah Wentworth^5^, Cecelia Wesseh^10^, Jimmy Whitworth^3^, Aurelie Wiedemann^1^, Wouter Willems^14^, Julian Williams, Barthalomew Wilson^10^, Njoh Wissedi^2^, Jayanthi Wolf^13^, Ian Woods^23^, Alie Wurie, Delphine Yamadjako^18^, Marcel Yaradouno^6^, Yazdan Yazdanpanah^1,7,8^, Zara Zeggani^6^.

**Affiliations**

^1^ French Institute for Health and Medical Research (INSERM), 75013 Paris, France

^2^ National Institute of Allergy and Infectious Diseases, Bethesda, MD, USA or under contract/subcontract to NIAID

^3^ London School of Hygiene & Tropical Medicine (LSHTM), London, UK

^4^ INSERM, Univ. Bordeaux, CIC 1401, EUCLID/F-CRIN clinical trials platform, UMR 1219, Bordeaux Population Health Research Center, CHU Bordeaux, Bordeaux, France

^5^ School of Public Health, University of Minnesota, Minneapolis, MN, USA

^6^ The Alliance for International Medical Action, Alima, B.P.15530 Dakar, Sénégal

^7^ AP-HP, Hôpital Bichat-Claude Bernard, Service de Maladies Infectieuses et Tropicales, Paris F-75018, France

^8^ REACTing, Institut Thématique Immunologie, Inflammation, Infectiologie et Microbiologie, Inserm, Paris, France

^9^ Centre National de Formation et de Recherche en Santé Rurale de Maferinyah, Maferinyah, Guinea

^10^ Partnership for Research on Ebola Virus in Liberia (PREVAIL), Monrovia, Liberia

^11^ College of Medicine and Allied Health Sciences (COMAHS), University of Sierra Leone, Freetown, Sierra Leone

^12^ University of Sciences, Technique and Technology of Bamako, Bamako, Mali

^13^ Merck & Co., Inc Whitehouse Station, New Jersey, USA

^14^ Janssen Vaccines and Prevention BV Leiden, The Netherlands

^15^ Département de Sociologie, FLSH, Université Cheikh Anta DIOP, Dakar Sénégal

^16^ Agence Nationale de Sécurité Sanitaire, Conakry, Guinea

^17^ Advanced BioMedical Laboratories, L.L.C., 1605 Industrial Hwy, Cinnaminson, NJ, USA

^18^ Leidos Biomedical Research, Inc. Frederick, MD 21704, USA

^19^ Battelle HQ, 505 King Avenue, Columbus, Ohio 43201

^20^ Centre pour le Développement des Vaccins, Ministère de la Santé, Bamako, Mali

^21^ University Clinical Research Center (UCRC)-SEREFO-Laboratory, University of Sciences, Techniques and Technologies of Bamako (USTTB), Bamako, Mali

^22^ Liberia Institute for Biomedical Research Ethics Committee/National, Monrovia, Liberia

^23^ Center for Vaccine Development and Global Health, University of Maryland School of Medicine, 685 West Baltimore Street Baltimore, MD 21201-1509, USA

^24^ INSP - Sorbonne Université - 4 place Jussieu, boîte courrier 840 - 75252 PARIS Cedex 05

^25^ École de santé publique de l’Université de Montréal, Montréal, Canada

**Appendix 4. Trial organization**


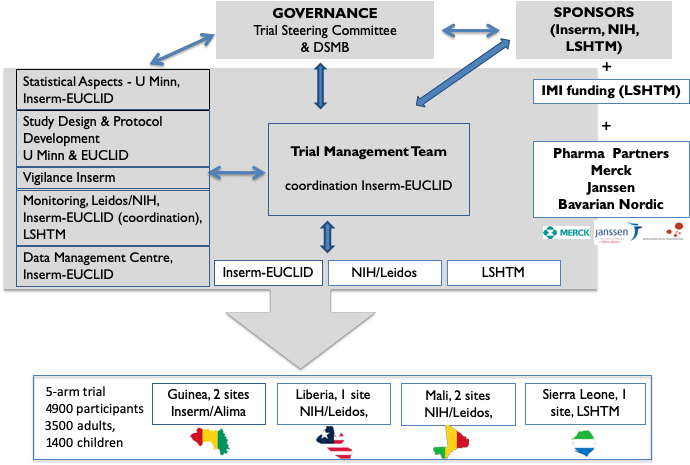


**Appendix 5 - Figure 1. Organization of the operations of the PREVAC trial**

**Appendix 5. Assent and consent forms**

**Partnership for Research on Ebola VACcination**

**(PREVAC)**

**ASSENT TO CONTINUE TO PARTICIPATE IN PREVAC**

**Coordinating Investigator:** Yazdan Yazdanpannah , Institut National de la Santé et de la Recherche Médicale (INSERM), France

**Country Principal**

**Investigators (PIs):** Abdoul Habib Beavogui, Guinea (Landreah and Maferinyah)

Cheick Mohamed Keita, Guinea (Landreah)

Pascal Niouma Millimouno, Guinea (Maferinyah)

Mark Kieh, Liberia

Stephen B. Kennedy, Liberia

Bailah Leigh, Sierra Leone

Mohamed H. Samai, Sierra Leone

Gibrilla Fadlue Deen, Sierra Leone

Seydou Doumbia, Mali (UCRC)

Mahamadou Diakite, Mali (UCRC)

Samba Sow, Mali (CVD)

Milagritos D. Tapia, Mali (CVD)

**Sponsors:** INSERM, France

National Institute of Allergy and Infectious Diseases, National Institutes of Health, United States

London School of Hygiene & Tropical Medicine (LSHTM), United Kingdom

**Sites:** Liberia, West Africa

Guinea, West Africa

Sierra Leone, West Africa

Mali, West Africa

#### what is THIS STUDY ABOUT?

You received an Ebola study vaccine on the first part of our PREVAC study. We would like to collect some more blood from you for up to 5 years after you received the study vaccine to keep looking at the effects of the Ebola vaccines. We would also like to better understand the long-term safety of the vaccines. No vaccines will be given in this part of the study. About 5,000 people will be taking part in this study in West Africa.

#### DO I HAVE TO continue to BE in this STUDY?

No. You can decide not to stay in the PREVAC study. Even if your parents want you to be in the study, you can still say no. You can talk to your family, friends, and doctor before you decide. Even if you want to stay in the study now, you can change your mind later and stop being in the study.

#### WHAT WILL HAPPEN TO ME IN THIS STUDY?

If you decide to remain in the PREVAC study, you will come back to the clinic once a year for a study visit. During the clinic visits we will take your temperature and weight, ask how you are feeling and if you have been sick during the year since your last visit.

Also, if you see a doctor or go to the hospital for an illness between your study visits, contact the study team as soon as possible after your illness to let them know. We need this information to help us better understand the long-term safety of the vaccines. Contact information for study participants in Liberia: Day time phone numbers 0881-255-266 or 0775-382-678, Night time phone numbers 0881-255-267 or 0775-382-679.

We will take a small amount of your blood for tests. These tests will tell us about the vaccines we are studying. A small needle will be put into a vein in your arm. We may save your blood to do tests in the future. Participants in Guinea and Sierra Leone will also have other tests conducted on their blood for more specific testing to see if infections like malaria or other blood infections change how the Ebola vaccine works.

Additionally, we would like to find out why people continue to participate in studies and what affects their decisions to participate in this research study. To do this, at each visit, we will also ask you some questions about things that may have affected your participation in this research study, such as questions about your family, how long it takes for you to get to the study visits, your emotional well-being, your experiences with Ebola, and how you feel about this research study. Answering these questions is optional. You can choose not to answer them and still be in this follow-up study.

You will have visits one time per year that will end after a total of five years from your first vaccination.

#### WHAT are the risks of BEING IN THIS STUDY?

This may hurt a little or feel like a mosquito bite. You may get a bruise.

If you agree to answer the optional questions, they may bring up memories about your experiences with Ebola, which may make you feel sad. You can refuse to answer any of these optional questions.

#### what are the benefits of being in this study?

Being in this study won’t help you, but it will help us learn how these vaccines work. This can help us and other scientists make vaccines to prevent Ebola and may in the future help people all over the world.

#### what will happen to YOUR blood SAMPLES AND PERSONAL INFORMATION?

We will keep your leftover blood samples and personal information for future research that may help us learn more about developing vaccines against Ebola, and for researchers who are investigating ways of improving the prevention or treatment of illnesses that are important to your country. You will not get any information from this. If you change your mind and decide you do not want us to store your blood samples or personal information anymore, please let us know. We will do our best to follow your wishes but cannot promise that we will always be able to destroy all your samples or personal information. For example, if your sample was already used, we would not be able to destroy it.

Your blood samples and personal information will be labeled with a code and not with your name. Your coded samples and personal information might be sent to other scientists, including scientists outside of your home country, for research. However, we will not share your name or other information that could be used to identify you. Your blood samples will not be sold. You will not be paid for any products that result from this research. The only risk of allowing us to store your samples or personal information would be an accidental release of your identity.

#### WHAT IF AN EBOLA VACCINE BECOMES APPROVED DURING THIS STUDY?

#### If an Ebola vaccine is licensed, it would be offered to you if there is an Ebola outbreak according to guidelines from the World Health Organization and the Ministry of Health in your country.

#### WHO CAN I TALK TO ABOUT THIS STUDY?

If you want to talk to anyone about this research study because you think you have been hurt by being part of the study, or you if have any other questions about the study, you should tell the study team: Day time phone numbers 0881-255-266 or 0775-382-678, Night time phone numbers 0881-255-267 or 0775-382-679.

Also, you can contact Gloria Mason coordinator of the National Research Ethics Board (Tel: +231-777-697-606/+231-886-697-606) to answer questions you may have about being part of this study and your rights as someone who is in a study.

If you have any questions at any time about this research study, you may ask someone on the study team.

Your participation in this study should not change your attitude toward public safety measures to prevent the spread of Ebola virus. In case of a new Ebola outbreak, you should continue to practice Ebola prevention behaviors.

The “Comité d'Evaluation Ethique de l'Inserm” (Inserm IRB; IRB00003888 and FWA00005831) initially approved this study on November 4th, 2016.

I agree to the storage of my blood samples for future research testing (after the end of the study) about infectious diseases in African countries.

By checking this box, I do **NOT** allow the storage of my blood samples for future research testing (after the end of the study) about infectious diseases in African countries.

If you agree to be in this study, please sign or put your fingerprint below.

Date: / /

Signature or fingerprint of minor participant *dd mon yyyy*

Printed name of minor participant

Date: / /

Signature of investigator *dd mon yyyy*

Printed name of investigator

*Complete if participant is illiterate:*

#### Witness to Consent Interview

On the date given next to my signature, I witnessed the consent interview for the research study named above in this document. I attest that the information in this consent form was explained to the subject, and the subject indicated that his/her questions and concerns were adequately addressed.

Date: / /

Signature of witness *dd mon yyyy*

Printed name of witness

**Partnership for Research on Ebola VACcination**

**(PREVAC)**

**CONSENT TO CONTINUE TO PARTICIPATE IN PREVAC**

**Coordinating Investigator:** Yazdan Yazdanpannah , Institut National de la Santé et de la Recherche Médicale (INSERM), France

**Country Principal**

**Investigators (PIs):** Abdoul Habib Beavogui, Guinea (Landreah and Maferinyah)

Cheick Mohamed Keita, Guinea (Landreah)

Pascal Niouma Millimouno, Guinea (Maferinyah)

Mark Kieh, Liberia

Stephen B. Kennedy, Liberia

Bailah Leigh, Sierra Leone

Mohamed H. Samai, Sierra Leone

Gibrilla Fadlue Deen, Sierra Leone

Seydou Doumbia, Mali (UCRC)

Mahamadou Diakite, Mali (UCRC)

Samba Sow, Mali (CVD)

Milagritos D. Tapia, Mali (CVD)

**Sponsors:** INSERM, France

National Institute of Allergy and Infectious Diseases (NIAID), National Institutes of Health (NIH), United States

London School of Hygiene & Tropical Medicine (LSHTM), United Kingdom

**Sites:** Liberia, West Africa

Guinea, West Africa

Sierra Leone, West Africa

Mali, West Africa

#### what is INVOLVED with ConTINUATION in PREVAC?

You recently finished participation in the first year of the PREVAC study of Ebola vaccines. We would like to collect some more blood from you for up to 5 years after you had received the study vaccination to continue looking at the long-term safety of the Ebola vaccines and to see how long we can measure their effects in the body. No vaccines will be given in this part of the study. About 5,000 people will be taking part in this study in West Africa.

In the rest of this consent document, ‘you’ can mean you or your child if you are thinking about allowing your child to remain in the study.

#### you can say “yes” or “no” to CONTINUE TO take part in this study.

You do not have to remain in the PREVAC study if you don’t want to. If you agree to remain in the study, we will ask you to sign this consent form. If you remain in the study, you can change your mind at any time and leave the study. If you decide not to remain in the study or to leave the study later, you will not lose any regular health care services you already are getting.

#### WHAT WILL HAPPEN DURING THE STUDY?

If you agree to remain in the PREVAC study, we will ask you to come to the clinic once a year for a study visit. During these visits, we will measure your temperature, and ask you questions about your health and how you were feeling in the year since your last visit. Children will have their height and weigh measured. For children between 1 and 5 years old, we will also measure the mid-arm circumference.

If you see a doctor or go to the hospital for an illness between your study visits, contact the study team as soon as possible after your illness to let them know. We need this information to help us better understand the long-term safety of the vaccines. Contact information for study participants in Liberia: Day time phone numbers 0881-255-266 or 0775-382-678, Night time phone numbers 0881-255-267 or 0775-382-679.

Blood will be taken by inserting a new, clean needle into a vein in your arm. We will use your blood for research tests that will help us learn more about how Ebola vaccines affect people. These tests are not part of your clinical care and are for research purposes only. Participants in Guinea and Sierra Leone will also have other tests conducted on their blood for more specific testing to see if infections like malaria or other blood infections change how the Ebola vaccine works.

Additionally, we would like to find out why people continue to participate in studies and what affects their decisions to participate in this research study. To do this, at each visit, we will also ask you some questions about things that may have affected your participation in this research study, such as questions about your family, how long it takes for you to get to the study visits, your emotional well-being, your experiences with Ebola, and how you feel about this research study. Answering these questions is optional. You can choose not to answer them and still be in this follow-up study.

You will have visits one time per year that will end after a total of five years from your first vaccination.

#### WHAT are the risks of BEING IN THIS STUDY?

Having your blood taken may cause pain or bruising where the needle goes in, lightheadedness, dizziness, fainting, extra bleeding, and, rarely, infection where the needle went in.

If you agree to answer the optional questions, they may bring up difficult emotions or memories about your experiences with Ebola, which may cause you stress or make you feel sad. We will also ask you some personal questions, including questions about your emotional health. You can refuse to answer any of these optional questions.

We will be careful to keep your study information private, but there is a small risk that someone not involved in the study could get this information.

#### what are the benefits of being in this study?

You will not receive any direct benefits from remaining in this study. Your participation in this study is important to learn how people respond to Ebola vaccines. It will help in the development of vaccines to prevent Ebola and may, in the future, help people all over the world.

#### what will happen to YOUR blood SAMPLES AND PERSONAL INFORMATION?

We will keep your leftover blood samples and personal information for future research that may help us learn more about developing vaccines against Ebola, and for researchers who are investigating ways of improving the prevention or treatment of illnesses that are important to your country. You will not get any information from this. If you change your mind and decide you do not want us to store your blood samples or personal information anymore, please let us know. We will do our best to follow your wishes but cannot promise that we will always be able to destroy all your samples or personal information. For example, if your sample was already used, we would not be able to destroy it.

Your blood samples and personal information will be labeled with a code and not with your name. Your coded samples and personal information might be sent to other scientists, including scientists outside of your home country, for research. However, we will not share your name or other information that could be used to identify you. Your blood samples will not be sold. You will not be paid for any products that result from this research. The only risk of allowing us to store your samples or personal information would be an accidental release of your identity.

#### WHAT IF AN EBOLA VACCINE BECOMES APPROVED DURING THIS STUDY?

#### If an Ebola vaccine is licensed, it would be offered to you if there is an Ebola outbreak according to guidelines from the World Health Organization and the Ministry of Health in your country.

#### Who will be able to see YOUR information?

We will keep your study information private. All files with information that could identify you will be kept in locked cabinets.

People responsible for making sure that the research is done properly may look at your study records. This might include people from the West African Agencies, the study sponsors (Inserm, NIH, and LSHTM) or the contract research organizations employed by the sponsors, the United States Food and Drug Administration, European regulatory bodies, and the drug companies that make the study vaccines (Janssen and Merck). All of these people are also obligated to keep your identity private.

#### WILL YOU BE COMPENSATED FOR BEING IN THE STUDY?

You will receive an inconvenience allowance for your time after each study visit.

#### WHAT IF YOU ARE HURT AS PART OF THE STUDY?

A study doctor will be available at all times while you are in the study to check on you. The doctor will give you any short-term medical care you need if you are hurt from the research blood draw being done in this study.

#### what else should i know about this study?

A description of this study will be on the internet at http://www.ClinicalTrials.gov. This website will not include information about you. At most, the website will include a summary of the results. You can search this website at any time.

The United States National Institutes of Health (NIH) researchers must tell the NIH at least yearly about any stock they own in the companies that make the study vaccines. All study investigators are also asked to do this. If you would like to get more information, you may ask your study team.

#### WHO CAN I TALK TO ABOUT THIS STUDY?

If you want to talk to anyone about this research study because you think you have been hurt by being part of the study, or if you have any questions about the study, you should tell the study team: Day time phone numbers 0881-255-266 or 0775-382-678, Night time phone numbers 0881-255-267 or 0775-382-679.

Also, you can contact Gloria Mason coordinator of the National Research Ethics Board (Tel: +231-777-697-606/+231-886-697-606) to answer questions you may have about being part of this study and your rights as someone who is in a study.

If you have any questions at any time about this research study, you may ask someone on the study team.

Your participation in this study should not change your attitude toward public safety measures to prevent the spread of Ebola virus. In case of a new Ebola outbreak, you should continue to practice Ebola prevention behaviors.

The “Comité d'Evaluation Ethique de l'Inserm” (Inserm IRB; IRB00003888 and FWA00005831) initially approved this study on November 4th, 2016.

I agree to the storage of my blood samples for future research testing (after the end of the study) about infectious diseases in African countries.

By checking this box, I do **NOT** allow the storage of my blood samples for future research testing (after the end of the study) about infectious diseases in African countries.

If you agree to be in this study, please sign or put your fingerprint below.

Date: / /

Signature or fingerprint of volunteer or guardian *dd mon yyyy*

Printed name of volunteer

Date: / /

Signature of investigator *dd mon yyyy*

Printed name of investigator

*Complete if participant is illiterate:*

#### Witness to Consent Interview

On the date given next to my signature, I witnessed the consent interview for the research study named above in this document. I attest that the information in this consent form was explained to the subject, and the subject indicated that his/her questions and concerns were adequately addressed.

Date: / /

Signature of witness *dd mon yyyy*

Printed name of witness

Attach PID bar-code label if not printed on the form:

**Appendix 6. Participants in the PREVAC trial**

The number of volunteers who participated in each version of the trial are shown in the figures below.

**Figure 6.1. Flow chart of the inclusion of the participants in the version 2.0 of the PREVAC trial**

**
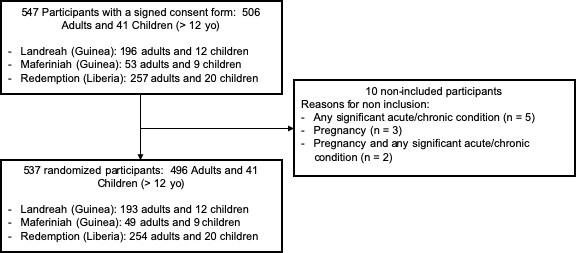
**

**Figure 6.2. Flow chart of the inclusion of the participants in the version 3.0 of the PREVAC trial**

**
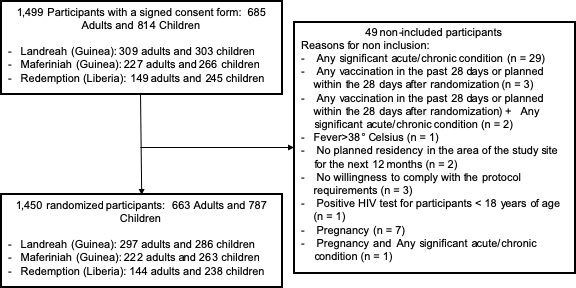
**

**Figure 6. 3. Flow chart of the inclusion of the participants in the version 4,0 of the PREVAC trial**

**
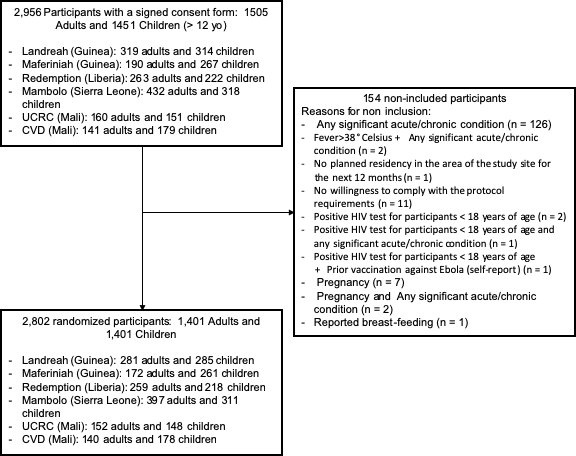
**

**Appendix 7. Enrollment of the participants in the PREVAC trial**

**
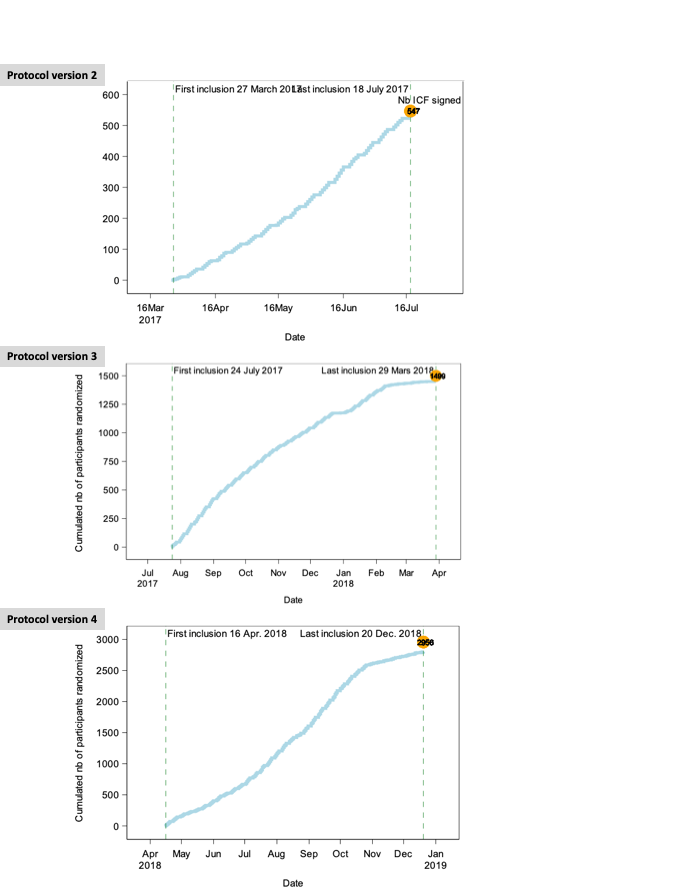
**

**Appendix 8. Baseline antibody data**

**Table 8. 1. Baseline laboratory measurements by country of the participants (adults and children) included in the PREVAC trial**

|  | | | | | | | | |
| --- | --- | --- | --- | --- | --- | --- | --- | --- |
|  | | | | | | | | |
|  | **Guinea (n=2328 )** | | **Liberia (n=1133 )** | | **Sierra Leone (n=707 )** | | **Mali (n=618 )** | |
|  |  | |  | |  | |  | |
|  | **Mean** | **SD** | **Mean** | **SD** | **Mean** | **SD** | **Mean** | **SD** |
| **Chemistries** |  |  |  |  |  |  |  |  |
| ALT (U/L) | 9.6 | 11.3 | 9.6 | 22.8 | 22.8 | 39.7 | 20.2 | 11.7 |
| AST (U/L) | 16.7 | 12.9 | 16.2 | 19.7 | 28.0 | 29.6 | 26.7 | 11.3 |
| Creatinine (mg/dl) | 0.81 | 0.26 | 0.81 | 0.25 | 0.55 | 0.20 | 0.66 | 0.27 |
| Potassium (mmol/L) | 4.2 | 0.4 | 4.1 | 0.4 | 4.1 | 0.4 | 4.3 | 0.4 |
|  |  |  |  |  |  |  |  |  |
| **Hematology** |  |  |  |  |  |  |  |  |
| White blood cells (x10³/μL) | 6.3 | 2.6 | 6.3 | 2.2 | 6.2 | 1.9 | 7.0 | 2.4 |
| Neutrophils (x10³/μL) | 2.7 | 1.1 | 2.8 | 1.3 |  |  | 2.8 | 1.3 |
| Lymphocytes (x10³/μL) | 2.8 | 1.7 | 2.5 | 1.0 | 2.9 | 1.2 | 3.1 | 1.4 |
| Eosinophils (x10³/μL) | 0.29 | 0.47 | 0.39 | 0.52 |  |  | 0.30 | 0.58 |
| Monocytes (x10³/μL) | 0.52 | 0.23 | 0.55 | 0.23 | 0.43 | 0.19 | 0.62 | 0.29 |
| Basophils (x10³/μL) | 0.10 | 0.09 | 0.09 | 0.05 |  |  | 0.09 | 0.05 |
|  | | | | | | | | |
| Hemoglobin (g/dl) | 12.6 | 1.7 | 13.1 | 1.9 | 12.3 | 1.7 | 12.3 | 2.0 |
| Hematocrit (%) | 38.7 | 5.0 | 39.6 | 5.1 | 37.0 | 5.1 | 37.0 | 5.6 |
| Platelets (x10³/μL) | 291.1 | 97.2 | 257.1 | 87.8 | 261.8 | 92.0 | 311.8 | 104.2 |
|  | | | | | | | | |
| Red blood cells (x10^6^/μL) | 4.8 | 0.6 | 4.9 | 0.6 | 4.6 | 0.6 | 4.6 | 0.6 |
| Red cell distribution width (%) | 13.6 | 1.8 | 12.9 | 1.4 | 14.3 | 1.3 | 13.5 | 1.9 |
| Mean corpuscular volume (fL) | 81.8 | 8.2 | 81.0 | 7.4 | 79.9 | 7.0 | 80.8 | 9.1 |
|  | | | | | | | | |
| Note - Lower limit of detection is imputed for undetectable results | | | | | | | | |
|  | | | | | | | | |

**
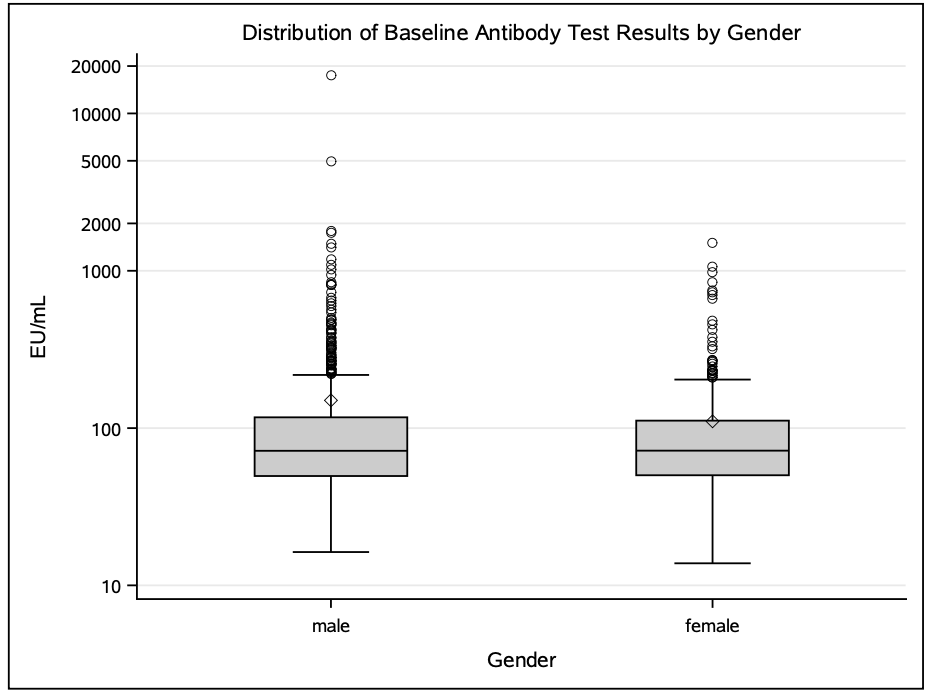
**

**Figure 8. 1. Distribution of baseline antibody response by gender**


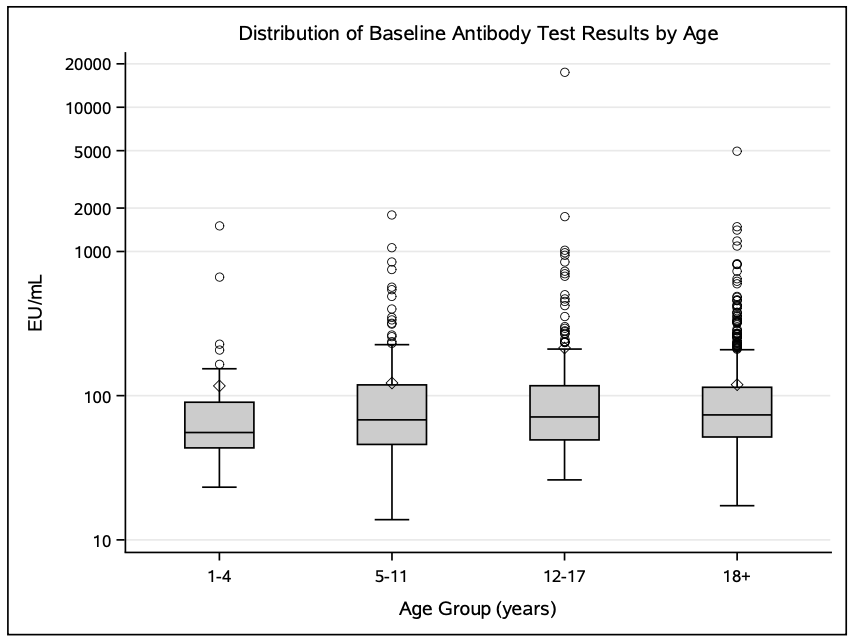


**Figure 8. 2. Distribution of baseline antibody response by age**
